# Orientation-Dependent Interaction between the Magnetic Plasmons in Gold Nanocups and the Excitons in WS_2_ Monolayer and Multilayer

**DOI:** 10.1021/acsnano.2c09099

**Published:** 2023-01-20

**Authors:** Ruoqi Ai, Xinyue Xia, Han Zhang, Ka Kit Chui, Jianfang Wang

**Affiliations:** † Department of Physics, The Chinese University of Hong Kong, Shatin, Hong Kong SAR999077, China; ‡ School of Materials Science and Engineering, Zhejiang Sci-Tech University, Hangzhou310018, China

**Keywords:** excitons, gold nanocups, magnetic plasmon resonance, plasmon−excition coupling, strong coupling, transition metal dichalcogenides

## Abstract

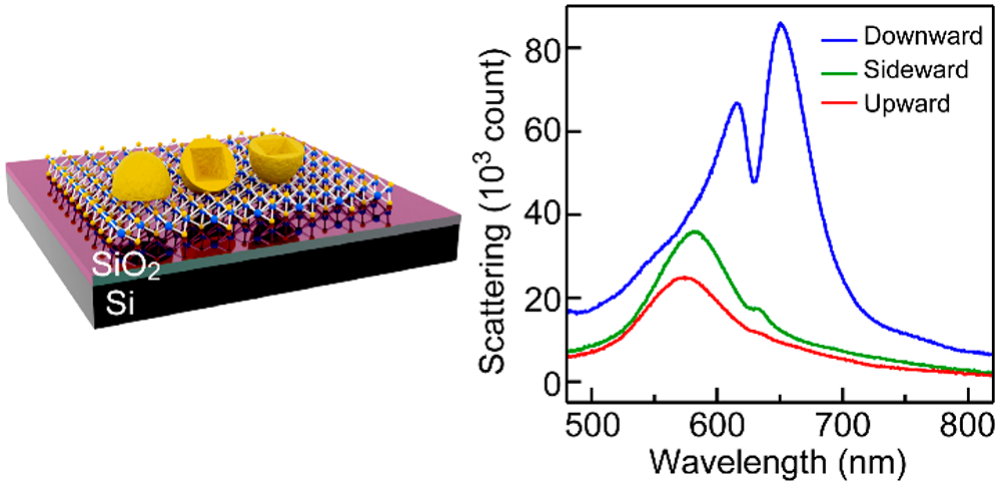

The integration of two-dimensional transition metal dichalcogenides with plasmonic nanostructures is extremely attractive for the investigation of the resonance coupling between plasmons and excitons, which offers a framework for the study of cavity quantum electrodynamics and is of great potential for exploring diverse quantum technologies. Herein we report on the coupling between the magnetic plasmons supported by individual asymmetric Au nanocups and the excitons in WS_2_ monolayer and multilayer. Resonance coupling with the strength varying from weak to strong regimes is realized by adjusting the orientation of the individual Au nanocups on WS_2_ monolayer. Different energy detunings between the magnetic plasmons and the excitons are achieved by varying the size of the Au nanocup. The Rabi splitting energies extracted at zero detuning are up to 106 meV. The anticrossing feature is observed in the measured scattering spectra and simulated absorption spectra, which indicates that the resonance coupling between the magnetic plasmons in the Au nanocup and the excitons in WS_2_ monolayer enters the strongly coupled regime. A dependence of the coupling strength on the layer number is further observed when the Au nanocups are coupled with WS_2_ multilayer. Our study suggests a promising approach toward the realization of different coupling regimes in a simple hybrid system made of individual Au nanocups and two-dimensional materials.

Light–matter interaction plays a significant role in various research fields, such as quantum communication,^1,2^ nonlinear optics,^3−5^ and polariton chemistry.^6−9^ It is usually difficult for an emitter in free space to interact strongly with photons because the light wavelength is typically much larger than the emitter size. Localized surface plasmon resonance has been well-known for its capability in concentrating incident light into nanoscale volumes and bringing large enhancement in light–matter interaction.^10,11^ A coupled harmonic oscillator model and a quantum mechanical Jaynes–Cummings model have been employed to reveal the coupling strength in a coupled plasmon–exciton hybrid system. In the quantum mechanical model, the coupling strength *g* can be determined according to
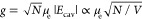
where *N* is the number of the excitons that are coupled to the cavity coherently, μ_e_ is the dipole moment strength of the exciton, and *E*_cav_ and *V* are the electric field and the mode volume of the cavity, respectively.^12,13^ Three different regimes can be observed. They are weak, intermediate, and strong coupling regimes. In the case of weak coupling, the scattering spectrum exhibits a single peak nearly located at the plasmon resonance. The quantum emitter decays with the spontaneous emission. The enhancement of the spontaneous emission can be calculated according to the Purcell factor.^14−17^ When the plasmon–exciton interaction reaches the intermediate coupling regime, a transparency dip is usually observed in the scattering spectrum. The dip is caused by Fano interference between the plasmon resonance and the quantum emitter resonance.^18^ In the strong coupling regime, the scattering peak is split into two separate peaks, which is known as Rabi splitting. In this regime, the energy exchange rate (Ω) between the nanocavity and the quantum emitter surpasses the dissipation rates of the corresponding plasmons and excitons. The energies in the excitons and photons are coupled, and dressed states are formed with intermixed light and matter characteristics.^19^ Strong coupling offers a powerful approach for fundamental investigations, such as Bose–Einstein condensation,^20,21^ superfluidity,^22,23^ quantum optoelectronics,^24^ and the quantum Hall effect.^25^

Two-dimensional transition metal dichalcogenides (TMDCs) with attractive features such as large binding energies, rich excitonic complexes, and flexible integration with other materials have become one of the most promising materials for the investigation of light–matter interaction.^26−28^ When the layer number of TMDCs is reduced to monolayer, they transform into direct-bandgap semiconductors with large exciton binding energies and exciton transition dipole moments that enable enhanced interaction of the dipole transitions with light.^29−31^ Plasmonic nanostructures can function as nanocavities to couple with the excitons in TMDC monolayers, giving rise to a variety of optical phenomena. Examples include strong coupling,^32−34^ plasmon-induced resonance energy transfer,^35^ and deep Fano resonance.^36^ The occurrence of these phenomena benefits from the strong dipole moments of the excitons in TMDC monolayers and the small mode volumes of plasmonic nanocavities. Such coupling has been observed in TMDCs interacting with different plasmonic nanostructures, such as nanobipyramids,^37^ nanorods,^38^ and nanoplates.^39^ These nanostructures with high symmetry possess only electric plasmon resonance. In contrast, our recently reported Au nanocups possess a highly asymmetric morphology and can support not only electric plasmon resonance but also magnetic plasmon resonance on an individual nanoparticle.^40−42^ They therefore provide additional possibilities for manipulating light–matter interaction between magnetic plasmon resonance and excitons.^43−45^ The magnetic plasmon resonance supported on Au nanocups is a transverse mode, which can be excited by the electric field aligned perpendicular to the symmetry axis of the cup. Under transverse excitation, electrons are collectively driven by the electric field of light to oscillate along the outside surface of the Au nanocup and form a circular loop current.^40^ Because the loop current is discontinuous at the opening region of the cup, very large electric and magnetic field enhancements are produced in the opening region. Moreover, the plasmonic properties of Au nanocups on substrates are orientation-dependent because of the special geometrical shape and the varied coupling strength of the nanocup with the substrate.^40^

In this work, we report on the resonance coupling in the (Au nanocup)-on-WS_2_ hybrid structures with an orientation-dependent behavior. Through control of the orientation and size of the nanocup, the magnetic plasmon energies of the nanocups are matched with the exciton transition energies of WS_2_ monolayer and multilayer. Different resonance coupling regimes ranging from weak to strong ones are achieved through control of the orientation of the Au nanocup on WS_2_ monolayer. The Au nanocups that are oriented with their opening against WS_2_ monolayer couple strongly with the latter because of the most intensive electromagnetic field enhancement that is imposed on WS_2_ monolayer in this orientation. Energy detuning between the magnetic plasmons and the excitons in WS_2_ monolayer is systematically varied by adjusting the size of the nanocup. The obtained Rabi splitting energy at zero detuning reaches 106 meV. Moreover, Au nanocups of varied sizes are deposited on the WS_2_ multilayer with their opening against the substrate. The coupling gets stronger as the number of WS_2_ layers is increased because of the participation of more excitons.

## Results and Discussion

The Au nanocup samples were synthesized following a previously described sacrificial templating method.^40^ It involves sequentially the synthesis of PbS nanooctahedra, asymmetric Au overgrowth, and the removal of the PbS templates through chemical etching (Figure S1a). The PbS nanooctahedra are uniform in size and shape. Scanning electron microscopy (SEM) imaging gave an average edge length of ∼70 nm (Figure S1b). The preprepared PbS nanooctahedron solution was injected into the growth solution with the Au precursor to produce Janus Au/PbS nanostructures (Figure S1c). The PbS nanooctahedra were finally etched away from the Janus Au/PbS nanostructures with HCl to yield the desired Au nanocup sample (Figure S1d).

Six Au nanocup samples with different sizes were prepared by varying the PbS seed amount (Figure S2). The sizes of the Au nanocups increase as the seed amount is reduced. The samples are labeled as 1–6 for convenience in the following discussion. All the samples exhibit relatively uniform sizes and relatively large opening with smooth opening edges, which is desired in the deposition of the Au nanocups to allow the opening of the nanocup to be oriented toward WS_2_ layer. Figure S3 shows the model of the Au nanocup illustrated in two different viewing directions. The model was used in description and finite-difference time-domain (FDTD) simulations. Four parameters, the cavity width *W*_cav_, cavity height *H*_cav_, cup width *W*_cup_, and cup height *H*_cup_, were used to define the sizes of the Au nanocup (Figure S3). The values of *W*_cav_, *W*_cup_, and *H*_cup_ were measured from the SEM images and are listed in Table S1. The value of *H*_cav_ can be calculated according to *H*_cav_ = *W*_cav_/√2. To better understand the plasmon modes of a single Au nanocup, FDTD simulations were carried out according to the measured dimensional parameters.

All of the six Au nanocup samples exhibit two resonance peaks, as revealed by their normalized extinction spectra (Figure S4a). The dominant extinction peak is originated from the transverse plasmon mode, which can be excited when the light polarization direction is aligned perpendicular to the symmetry axis. This mode possesses the characteristic of magnetic plasmon resonance.^40,41^ The weak shoulder at the higher-energy side arises from axially polarized excitation, which can mainly be attributed to an electric dipole mode.^40,41^ As the nanocup gets larger, the peak arising from the magnetic plasmon resonance shifts in the red direction gradually and the peak arising from the electric plasmon resonance becomes clearer. We carried out FDTD simulations using the size parameters of the Au nanocup samples given in Table S1. The incidence direction of the excitation light was set to be along the axial direction (*z* axis), with the electric field polarized along the transverse direction (*x* axis) (Figure S4b). The magnetic dipole mode is excited, and the simulated spectra (Figure S4c) are in good agreement with the corresponding measured ones. In this study, only the magnetic dipole mode was considered because the electric plasmon mode is much weaker in intensity than the magnetic one.^40^ The magnetic plasmon mode is dominant in the interaction of the cup plasmons with the excitons of WS_2_ layers.

The WS_2_ sample was prepared using a previously reported physical vapor deposition method in a tube furnace (Figure S5a).^46^ The produced WS_2_ nanosheet has a triangular shape (Figure S5b). Its thickness was measured by atomic force microscopy (AFM) to be ∼0.75 nm (Figure S5c). Two prominent peaks located at 351 and 419 cm^–1^ were detected on the WS_2_ sample in Raman measurements (Figure S5d). They can be ascribed to the E_2g_^1^ and A_2g_ vibrational modes of WS_2_ monolayer.^47^ All of these characterization results indicate the successful growth of WS_2_ monolayer.

**Figure 1:**
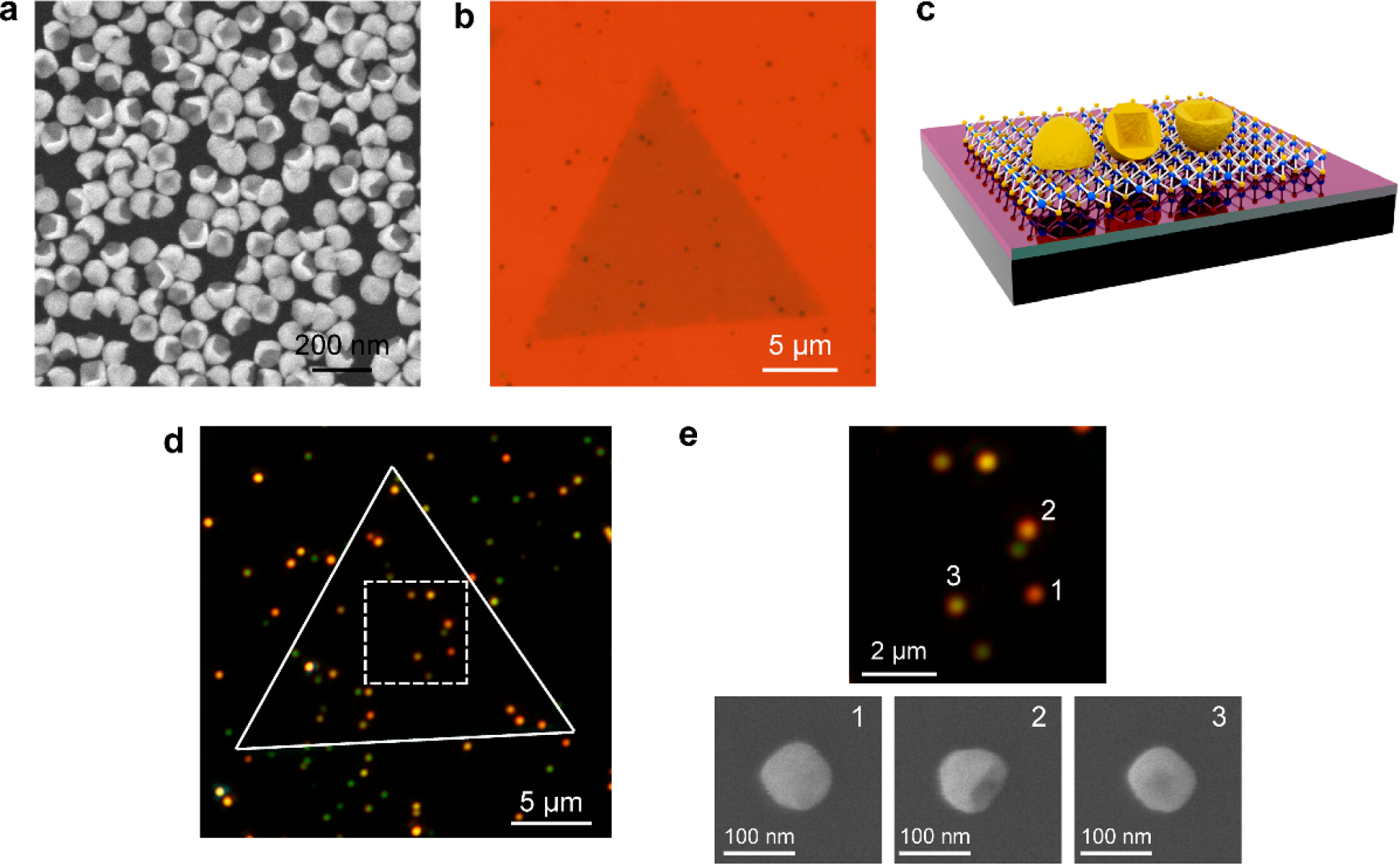
Hybrid structures made by depositing the Au nanocups onto the WS_2_ monolayer. (a) Representative SEM image of the nanocups. (b) Bright-field optical image of a triangular piece of WS_2_ monolayer deposited with the Au nanocups. (c) Schematic illustration of the (Au nanocup)-on-(WS_2_ monolayer) hybrid structures with the Au nanocups oriented in the three different directions. (d) Dark-field scattering image of the hybrid structures. The imaging region is the same as (b). The white triangle indicates the WS_2_ monolayer. (e) Dark-field image of the area marked with the white dashed box in (d) (upper) and SEM images of three Au nanocups in different orientations (lower).

To study the interaction of the plasmons with the excitons, hybrid structures made of the WS_2_ monolayer and the Au nanocups were constructed. The Au nanocup sample 3 was employed. Figure 1a shows a representative SEM image of the Au nanocups. The as-prepared Au nanocups were stabilized with cetyltrimethylammonium bromide (CTAB) in aqueous solutions. The excess CTAB was removed by centrifugation and redispersion in water twice. The nanocups capped with the residual CTAB were then sparsely deposited onto the WS_2_ monolayer by drop-casting. Under a bright-field optical microscope, the WS_2_ monolayer appears in a triangular shape and the individual Au nanocups appear as small black dots (Figure 1b). The gap distance between the Au nanocup and the WS_2_ monolayer surface is estimated to be ∼1 nm according to the thickness of the CTAB adsorbate.^48^ After deposition, the Au nanocups are oriented approximately in three different directions on the WS_2_ monolayer, i.e., with the opening downward (against the substrate), sideward (the side surface of the Au nanocup in contact with the WS_2_ monolayer), and upward (opposite to the substrate), as schematically illustrated in Figure 1c. Figure 1d shows the dark-field scattering image of the formed (Au nanocup)-on-(WS_2_ monolayer) hybrid structures. The imaging region is the same as Figure 1b. The WS_2_ monolayer is invisible under dark-field optical microscopy and indicated with the white triangle in the image. The dark-field scattering patterns of the Au nanocups appear as solid bright spots with different colors, as shown by the zoomed-in scattering image in Figure 1e. The different colors can be ascribed to the size distribution and the different orientations of the nanocups on the flat substrate. Three representative examples with different colors and their corresponding SEM images are shown in Figure 1e. When the cup opening is toward the WS_2_ layer, the dark-field scattering pattern shows a uniform dark red color. In comparison, the patterns of the Au nanocups in the other orientations exhibit nonuniform colors. The same orientation-dependent dark-field scattering patterns can also be observed from the Au nanocups deposited on SiO_2_ substrates without any WS_2_ layer (Figure S6). We can therefore easily distinguish the downward orientation of the Au nanocups from their dark-field scattering patterns.

**Figure 2:**
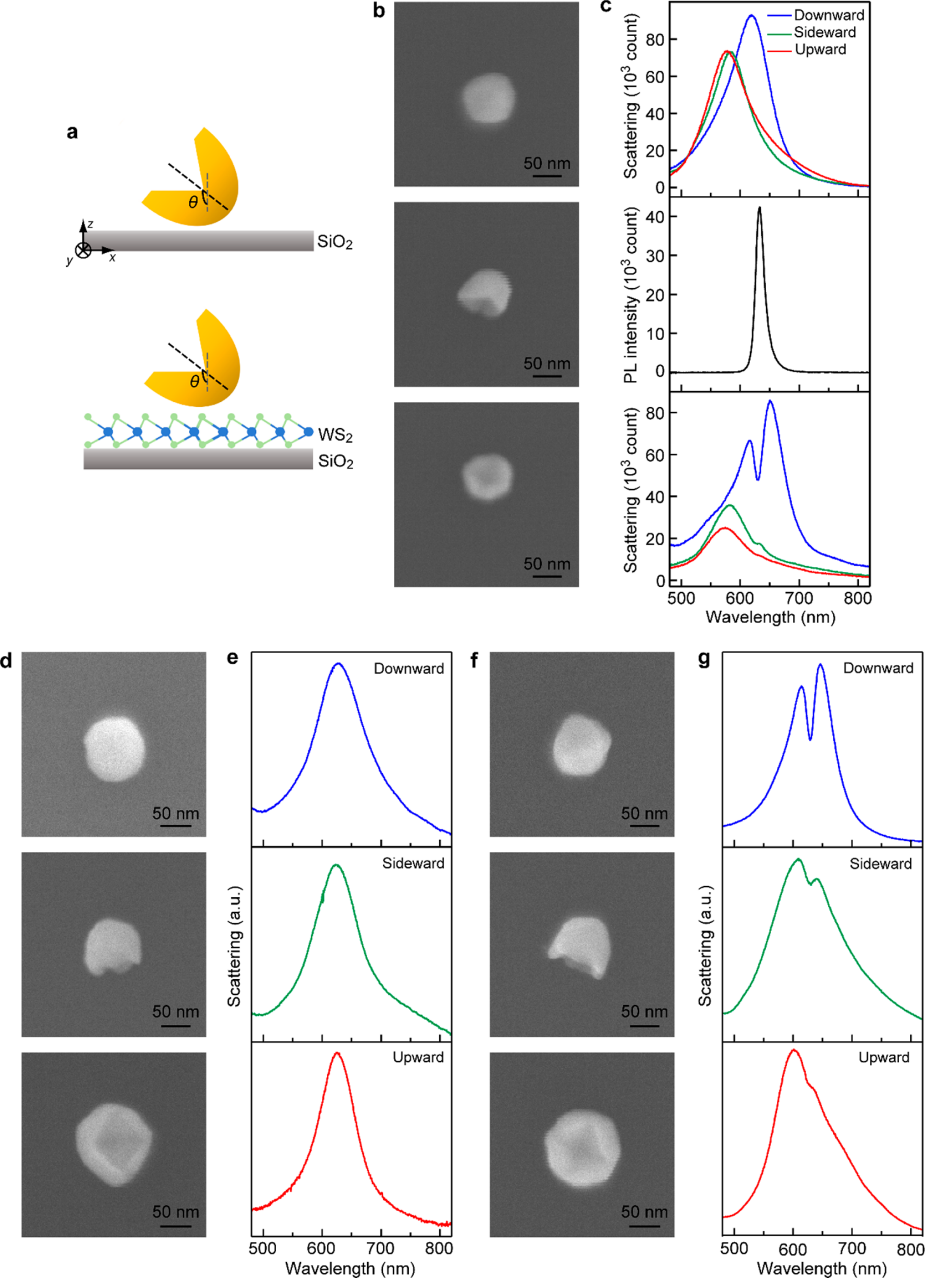
Plasmon–exciton interaction between the Au nanocups and the WS_2_ monolayer. (a) Schematics of a Au nanocup on a SiO_2_ substrate (upper) and a Au nanocup on WS_2_ monolayer supported on a SiO_2_ substrate (lower). (b) SEM images showing three representative (Au nanocup)-on-(WS_2_ monolayer) hybrid structures with the nanocups taking the three typical orientations. (c) Upper: scattering spectra measured from three Au nanocups that exhibited the three typical orientations on SiO_2_ substrates (upper). Middle: PL spectrum measured on a grown WS_2_ monolayer. Lower: scattering spectra measured from the three (Au nanocup)-on-(WS_2_ monolayer) hybrid structures displayed in (b). The excitation laser was at 488 nm and had an optical power of 0.225 mW. (d) SEM images showing three differently sized Au nanocups deposited on SiO_2_ substrates. The nanocups assume the three typical orientations. (e) Scattering spectra of the Au nanocups with nearly the same plasmon wavelengths on SiO_2_ substrates. (f) SEM images of three differently sized Au nanocups on WS_2_ monolayer. (g) Scattering spectra measured from the three (Au nanocup)-on-(WS_2_ monolayer) hybrid structures. The sizes of the Au nanocups in (f) are correspondingly approximately equal to those with the same orientations in (d).

Single-particle dark-field scattering measurements were carried out to study the interaction of the Au nanocups in the three different orientations with the WS_2_ monolayer. The interaction of the individual nanocups with the WS_2_ monolayer shows strong orientation dependence. Figure 2a shows the schematics of a nanocup on a SiO_2_ substrate (upper image) and on a piece of WS_2_ monolayer supported by a SiO_2_ substrate (lower image), respectively. θ is the angle formed by the symmetry axis of the nanocup and the *z* axis, which is normal to the substrate and points upward. The three typical orientations were considered, i.e., the nanocup in the downward orientation (θ ≈ 0°), the nanocup in the sideward orientation (θ ≈ 90°), and the nanocup in the upward orientation (θ ≈ 180°). The SEM images of the (Au nanocup)-on-(WS_2_ monolayer) hybrid structures with the nanocups in the three orientations are shown in Figure 2b. The shown Au nanocups are from sample 3 with similar sizes (Figure S2c). The scattering spectra of the Au nanocups deposited on SiO_2_ substrates in the three different orientations are displayed in Figure 2c (upper panel). As the cup orientation is changed from the upward to downward, the scattering peak first shows a slight redshift in the sideward orientation and then a large redshift in the downward orientation. The Au nanocup with its opening toward the substrate exhibits a plasmon peak centered at ∼630 nm, whose energy matches the exciton energy of the WS_2_ monolayer (Figure 2c, middle panel). The grown WS_2_ monolayer exhibits a single pronounced PL peak at 631 nm, which can be ascribed to the direct bandgap emissions of WS_2_ monolayer.^46^ For the Au nanocups coupled to the WS_2_ monolayer in the downward orientation, mode splitting was clearly observed in the scattering spectrum (Figure 2c, lower panel). On the contrary, such splitting was difficult to be observed for the Au nanocups in the other orientations, which suggests weak coupling. These observations can be ascribed to either the wavelength mismatch or the different coupling mechanisms determined by the nanocup orientation, which will be discussed below.

We fixed the plasmon wavelengths of differently oriented Au nanocups to be resonant with that of the excitons in WS_2_ monolayer by synthetically adjusting the sizes of the nanocup. In this context, the impact of the nanocup orientation on the plasmon–exciton coupling strength can be revealed clearly. Three Au nanocup samples of different sizes were selected. The scattering spectra of three pairs of Au nanocups with different sizes were measured and compared (Figure 2d–g). For each pair, one nanocup is on SiO_2_ (Figure 2d) and the other is on SiO_2_-supported WS_2_ monolayer (Figure 2f). The Au nanocups in the same orientation are similar in size. When the nanocup opening is toward the WS_2_ monolayer, mode splitting in the scattering spectrum was observed (Figure 2g, upper panel). When the Au nanocups are in the sideward and upward orientations, such splitting was difficult to be observed in the scattering spectra (Figure 2g, middle and lower panels). These results eliminate the doubt that the coupling strength difference in Figure 2c might be caused by wavelength mismatch and thereby confirm that the Au nanocups in the downward orientation can interact more strongly with the excitons in WS_2_ monolayer. The Au nanocups in the downward orientation were therefore considered in the following experiments.

The scattering spectra of the Au nanocups deposited on SiO_2_ substrates in the three different orientations were simulated by the FDTD method to understand the spectral differences (Figure S7a). In the simulation setting, the excitation light was normal to the substrate along the negative *z* axis and polarized along the *x* axis. The direction of the plasmonic electric field relative to that of the exciton transition dipole moment plays a crucial role in plasmon–exciton coupling.^33,34,49^ The bright exciton dipole is in-plane-oriented for WS_2_ monolayer and can greatly interact with the plasmonic electric field in the same or a parallel plane. The in-plane (*x*–*y* plane) electric field enhancement induced by the Au nanocup is therefore mainly concerned in the following discussion. For all electric and magnetic enhancement considered below, the *x*–*y* contours were taken on the middle plane in between the Au nanocup and the substrate, and those in the *x*–*z* plane were taken from the plane with the maximal electromagnetic field enhancement. When the nanocup is in the downward orientation, normally incident light can excite the magnetic plasmon resonance mode. Moreover, the excited plasmon resonance can interact with its own image charges generated in the substrate, resulting in a pronounced redshift of the scattering peak. This is consistent with the experimental result (Figure 2c, upper panel). In the other two orientations, although the magnetic plasmon mode can be excited as well, the much smaller shifts of the plasmon band indicate that the nanocup cannot couple efficiently with the substrate. We further simulated and analyzed the field enhancement and charge distribution contours of the Au nanocup assuming the three typical orientations at the corresponding resonance peaks to ascertain their plasmonic properties. When the Au nanocup sits with its opening toward the substrate, the electric field is concentrated in the region adjacent to the substrate (Figure S7b,c, top panels), and the magnetic field is intensively enhanced inside the nanocup close to the opening (Figure S7d,e, top panels). The magnetic field enhancement is largely generated at the opening region along the *y* axis. It results from the loop current in the Au nanocup.^50^ Such an electromagnetic field distribution can cause the Au nanocup to interact strongly with the substrate. The charge distribution shows the feature of a dipole mode (Figure S7f, top panel). When the nanocup is in the sideward orientation, the electric field is focused at the opening of the Au nanocup, and the charge distribution also exhibits the feature of a dipole mode (Figure S7b,c,f, middle panels). The magnetic field is enhanced inside the Au nanocup as well as in the region adjacent to the substrate (Figure S7d,e, middle panels). The enhancement is lower than that of the Au nanocup in the downward orientation. Similar results were also observed in the case where the Au nanocup was in the upward orientation (Figure S7b–f, bottom panels). These results suggest that the Au nanocup in the downward orientation can strongly couple with the substrate and generate large electric and magnetic field enhancements near the contact area.

**Figure 3:**
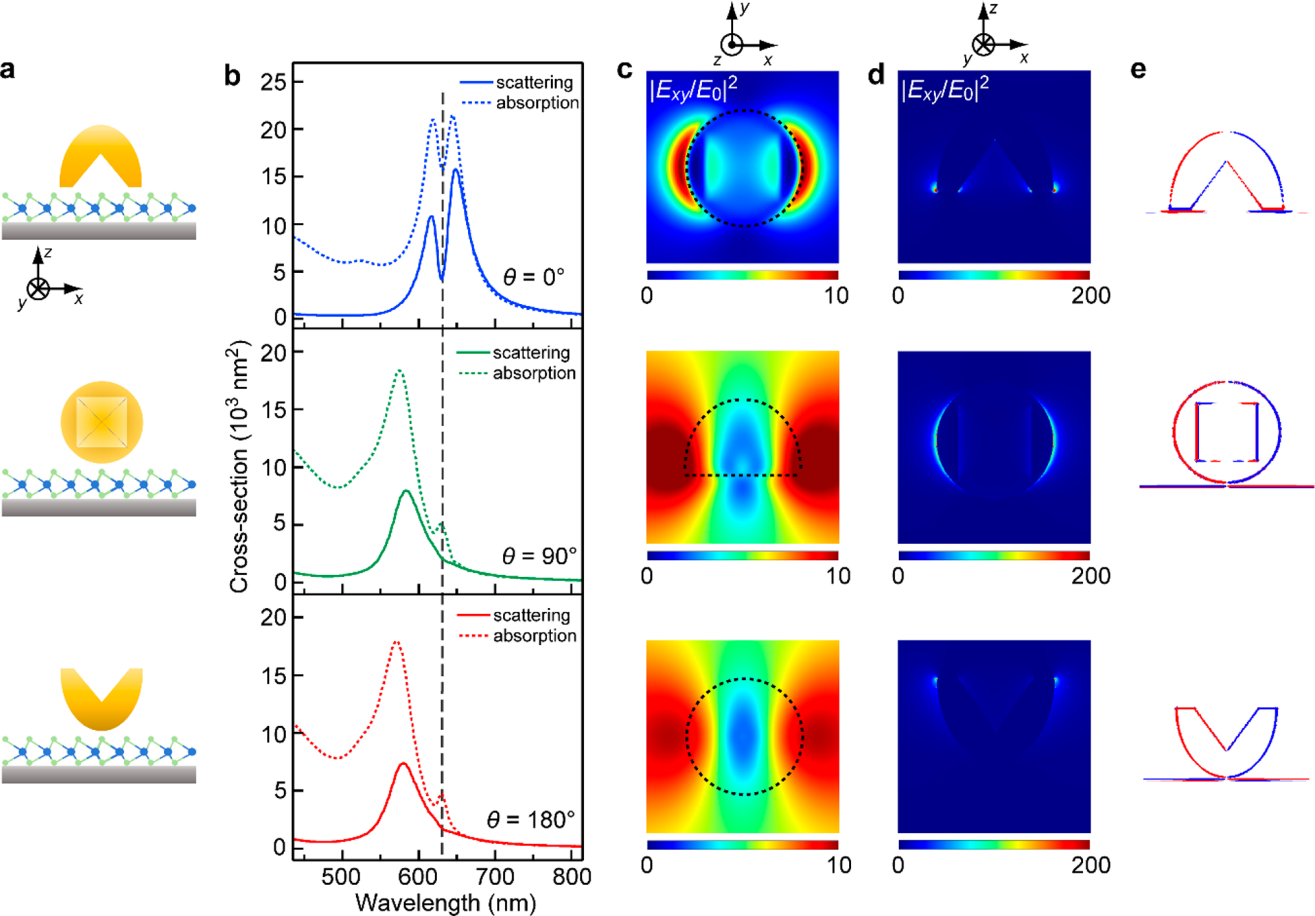
Simulation of the single Au nanocup deposited on WS_2_ monolayer. (a) Schematics of the (Au nanocup)-on-(WS_2_ monolayer) hybrid structures with the nanocup assuming the three typical orientations. (b) Simulated scattering and absorption spectra of the three (Au nanocup)-on-(WS_2_ monolayer) hybrid structures. (c, d) Electric field enhancement contours taken on the *x*–*y* (c) and *x*–*z* (d) planes. The dashed lines indicate the Au nanocups. (e) Charge distribution contours on the *x*–*z* plane. All the contours were simulated at the wavelength of 631 nm.

The Au nanocup assuming the three typical orientations and coupled with WS_2_ monolayer was also investigated by FDTD simulations (Figure 3a). The same excitation configuration was employed, where the incident light was normal to the substrate and polarized along the *x* axis. For all electric and magnetic enhancements below, the *x*–*y* plane was taken from the center of the dielectric layer between the Au nanocup and the substrate, and the *x*–*z* plane was taken from the plane with the maximal electromagnetic field enhancement. When the Au nanocup is in the downward orientation, the magnetic plasmon resonance redshifts and couples with the excitons in the WS_2_ monolayer, leading to mode splitting in the scattering spectrum (Figure 3b, top panel, blue solid line). The same splitting is also observed in its absorption spectrum (Figure 3b, top panel, blue dashed line), suggesting that the interaction between the excitons and the magnetic plasmon resonance does not result from a Fano resonance or enhanced absorption and that the system starts to enter the strong coupling regime.^51^ In the sideward and upward orientations, the coupling is very weak. The dips in the scattering spectra are very shallow but the absorption spectra exhibit clear peaks at the same wavelength (Figure 3b, middle and bottom panels), indicating that there exists energy transfer between the magnetic plasmon resonance and the excitons. We further considered the scattering dip of these systems at the wavelength of 631 nm and simulated and analyzed their electric field enhancement and charge distribution contours. For the downward orientation, the electric field can be strongly enhanced in the interface region between the nanocup and the WS_2_ monolayer, resulting in strong interaction with the excitons in the WS_2_ monolayer (Figure 3c,d, top panels). In contrast, the electric field enhancement for the nanocup assuming the other two orientations is weak, which leads to weak coupling (Figure 3c,d, middle and bottom panels). The strong plasmon–exciton interaction achieved for the downward orientation is enabled by the magnetic plasmon resonance, which can cause the electric field in the WS_2_ layer to be strongly enhanced and thus act on the in-plane WS_2_ excitons.^52^ Moreover, when the opening of the Au nanocup points to the WS_2_ layer, the charge distribution at the scattering dip shows the quadrupole mode characteristic (Figure 3e, top panel) due to strong interaction between the plasmons and the excitons, which is different from that of the case without any WS_2_ layer (Figure S7f, top panel). The strongly coupled excitons can affect the plasmon mode through near-field proximity interaction, resulting in the change from the dipole mode supported on the Au nanocup in the downward orientation to the quadrupole mode.^53^ In contrast, the charge distributions for the Au nanocup in the other two orientations show the feature of the dipole mode (Figure 3e, middle and bottom panels), remaining the same as that without the WS_2_ monolayer (Figure S7f, middle and bottom panels). Since no clear mode splitting can be observed, the plasmon–exciton interaction is weak in these two cases. To gain further insight into the plasmon–exciton interaction in the three orientations, we further simulated the electric/magnetic field enhancement and charge distribution contours at their scattering peaks/dips (Figures S8–S10). These simulation results reveal again that the nanocup in the downward orientation can greatly focus electromagnetic field into the region beneath the nanocup and thus enhance the plasmon–exciton interaction, while the nanocup in the other two orientations does not possess this capability. Taken together, interactions from weak to strong are achieved by varying the orientation of the Au nanocup on WS_2_ monolayer.

In addition to the three typical orientations, we also carried out FDTD simulations on the Au nanocups supported on Si/SiO_2_ substrates in other orientations, taking normal incident light with *x*- and *y*-polarization into consideration. Under *x*-polarized excitation (Figure S11a), for the nanocup with θ = 0° and 180°, only the transverse mode can be excited. When θ = 90°, only the axial mode can be excited. In other cases (θ ≠ 0°, 90°, or 180°), both the transverse and axial modes can be excited. Only one scattering peak in the total scattering spectrum is observed due to the closeness of the transverse and axial modes. The redshift for θ = 0° is due to the coupling with the substrate (Figure S11b), as mentioned above. When the WS_2_ monolayer is present (Figure S11e), only the case of θ = 0° shows a clear mode splitting (Figure S11f). Under *y*-polarized excitation, no matter in which direction the nanocup is oriented, only the transverse mode can be excited (Figure S11c). When the orientation of the nanocup is changed from θ = 180° to θ = 0°, the scattering peak shows slight redshifts at the beginning and then displays an abrupt redshift when θ becomes 0° (Figure S11d). When the WS_2_ monolayer is included (Figure S11g,h), mode splitting can be observed for all cases, the strongest of which is the nanocup with the opening exactly against the substrate. This variation trend is generally in agreement with that observed in our experiments.

**Figure 4:**
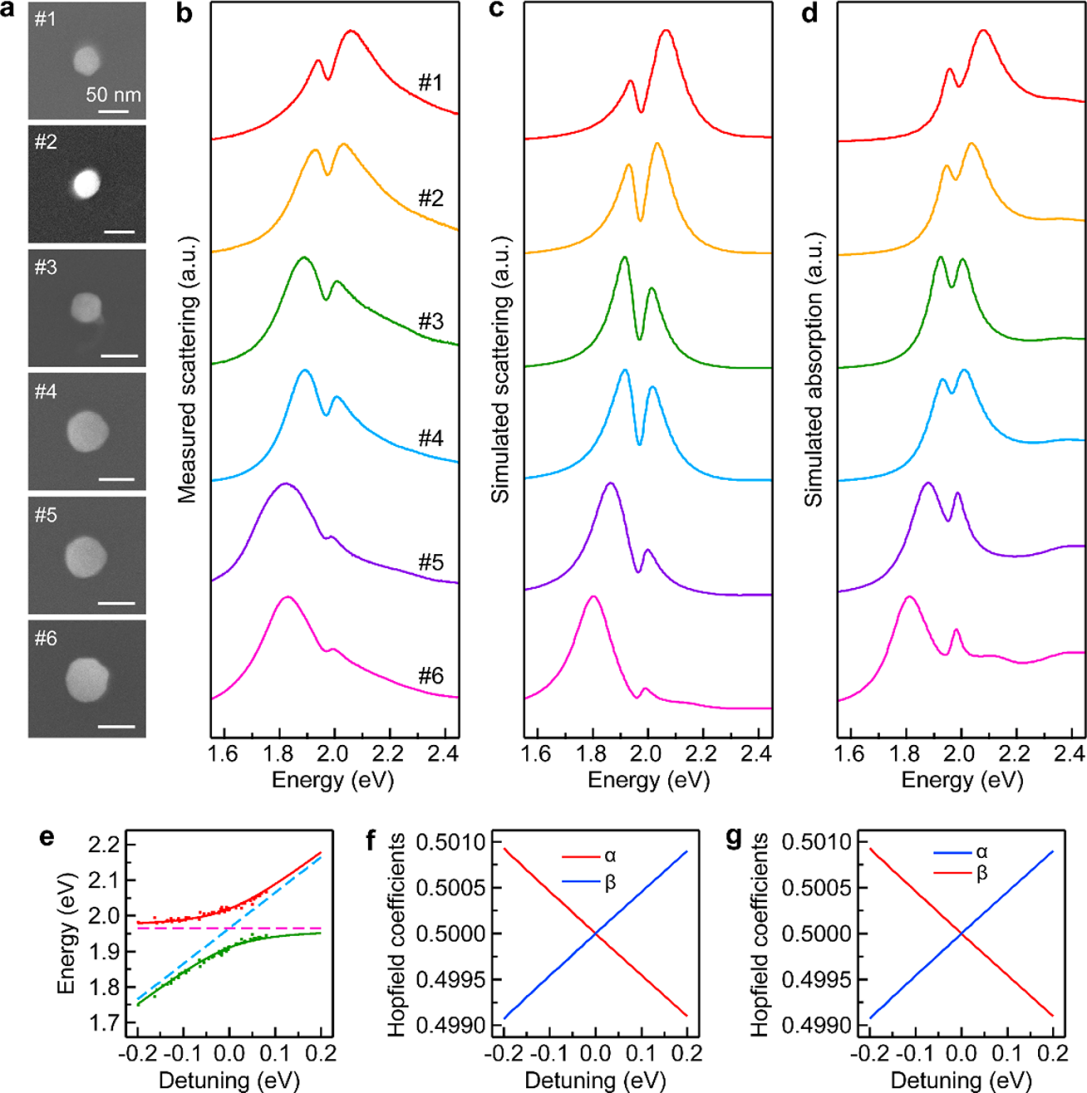
Resonance coupling in the (Au nanocup)-on-(WS_2_ monolayer) hybrid structures with the nanocup in the downward orientation. (a) SEM images of the six representative hybrid structures. (b–d) Measured scattering spectra (b) and simulated scattering (c) and absorption (d) spectra of the hybrid structures made of differently sized Au nanocups. The size parameters determined from the SEM images in (a) were employed in the FDTD simulations. (e) Peak energies retrieved from the scattering measurements (red and green dots) as functions of the detuning energy between the plasmons and excitons. The two solid lines in red and green were obtained from fitting according to the coupled oscillator model. The two dashed lines in magenta and blue represent the uncoupled exciton and plasmon energies, respectively. (f) Hopfield coefficients α and β (|α|^2^ + |β|^2^ = 1) of the upper branch. α and β are the weighting coefficients of the excitons and plasmons, respectively. (g) Hopfield coefficients of the lower branch.

Both the experimental and simulated results show that only the downward orientation can reach the strongly coupled regime. We therefore focused below on the downward orientation and studied the effect of the energy detuning on strong coupling. The six Au nanocup samples 1–6 (Figure S2) were deposited on WS_2_ monolayer and subjected to the scattering measurements (Figure 4a,b and Figure S12). The magnetic plasmon mode is progressively varied from high to low energies, crossing the transition energy of the exciton at 1.965 eV. The mode splitting occurs in all scattering spectra and changes along with the plasmon–exciton detuning (Figure 4b), with the detuning referring to the energy difference between the plasmons and excitons. The split mode at the lower energies dominates when the magnetic plasmon mode is smaller than the exciton transition in energy. At zero detuning, the dip arising from the mode splitting becomes the deepest, indicating the emergence of photon–matter hybrid states. When the magnetic plasmon resonance energy is larger than the exciton transition energy, the split higher-energy mode becomes stronger than the lower-energy one. The simulated scattering spectra show the similar evolution behavior in the spectral shape (Figure 4c). However, the peak splitting in scattering can result from electromagnetically induced transparency or enhanced absorption.^37,39,51^ To examine the origin of the coupled state in our system, we also simulated the absorption spectra (Figure 4d). When the hybrid system is in the weak coupling regime, there exists only a single peak in the absorption spectrum. The peak is caused by the electromagnetically induced transparency and located at the plasmon resonance position of the metal nanoparticle. In the case of the strongly coupled system, the absorption spectrum shows large splitting that is similar to the scattering spectrum due to the enhanced absorption (Figure 4d). This feature indicates that our system is in the strongly coupled regime.^37,39,51^

To further ascertain which coupling regime our (Au nanocup)-on-(WS_2_ monolayer) hybrid structures belong to, we extracted the split peak positions of a series of scattering spectra with different energy detunings (Figure 4e). Two distinct branches featuring the anticrossing characteristic were observed. They represent the upper and lower energy modes. The occurrence of the anticrossing behavior typically indicates that the interaction in a plasmon–exciton system is in the strong coupling regime.^54−56^ The coupling strength in our system was estimated using the coupled harmonic oscillator model^12,54^
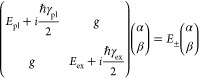
1where *E*_pl_ is the uncoupled plasmon energy, *E*_ex_ represents the uncoupled exciton transition energy, γ_pl_ is the plasmonic dissipation rate, γ_ex_ represents the excitonic dissipation rate, *g* is the coupling strength, and α and β are the Hopfield coefficients.^12,33,56,57^ α and β satisfy the condition |α|^2^ + |β|^2^ = 1. They account for the linear combination of the plasmonic and excitonic states. For simplicity, the plasmonic and excitonic dissipations were ignored. The expressions for the eigenvalues and coupling strength were therefore derived as
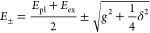
2
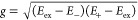
3where δ = *E*_pl_ – *E*_ex_ is the detuning energy between the plasmons and excitons. When *E*_pl_ = *E*_ex_, the Rabi splitting energy is ℏΩ = 2*g*. Moreover, the two branches obtained from both experiments and simulations for the coupled system were fitted according to the *E*_±_ expressions. *E*_ex_ was taken as 1.96 eV according to the PL result (Figure 2c). The anticrossing fitting of the experimental results according to eq 2 (Figure 4d, solid curves) gave a value of ℏΩ = 2*g* = 106 meV for the Rabi splitting energy of the hybrid nanostructures when the plasmons and the excitons are in perfect resonance. Several different criteria have been proposed to classify the coupling regime for a plasmon–exciton system. One criterion that has been proposed^12,58−60^ for strong coupling is 2*g* > (γ_pl_ – γ_ex_)/2. Another stricter criterion^13,34,37−39^ for strong coupling is 2*g* > (γ_pl_ + γ_ex_)/2. In this study, the plasmon line width of the nanocup was extracted to be 210 meV. The exciton line width was found to be 40 meV. The Rabi splitting energy in this system is larger than (γ_pl_ – γ_ex_)/2 and smaller than (γ_pl_ + γ_ex_)/2. The regime of strong coupling is also often accompanied by a distinct mode splitting and an anticrossing feature in the absorption spectra of the coupled system, which have been shown in Figures 3b and 4d for our system.^37,38,51^ Although our system does not fulfill the strict criterion for strong coupling (2*g* > (γ_pl_ + γ_ex_)/2) in a clear-cut manner, the Rabi splitting energy approaches closely to (γ_pl_ + γ_ex_)/2. Therefore, the coupling in the downward orientation in our (Au nanocup)-on-(WS_2_ monolayer) hybrid system is very close to the beginning of strong coupling.^60,61^ Moreover, the Hopfield coefficients were calculated to estimate the components of the excitons and plasmons in the plexcitonic system. The equations for the calculation are expressed as
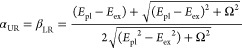
4
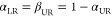
5where UR and LR represent the upper and lower resonance branches, respectively. At zero detuning, the Hopfield coefficients for the upper and lower branches are close to 0.5 (Figure 4f,g), indicating that these two states are coupled to form fully hybridized plexcitonic states.

**Figure 5:**
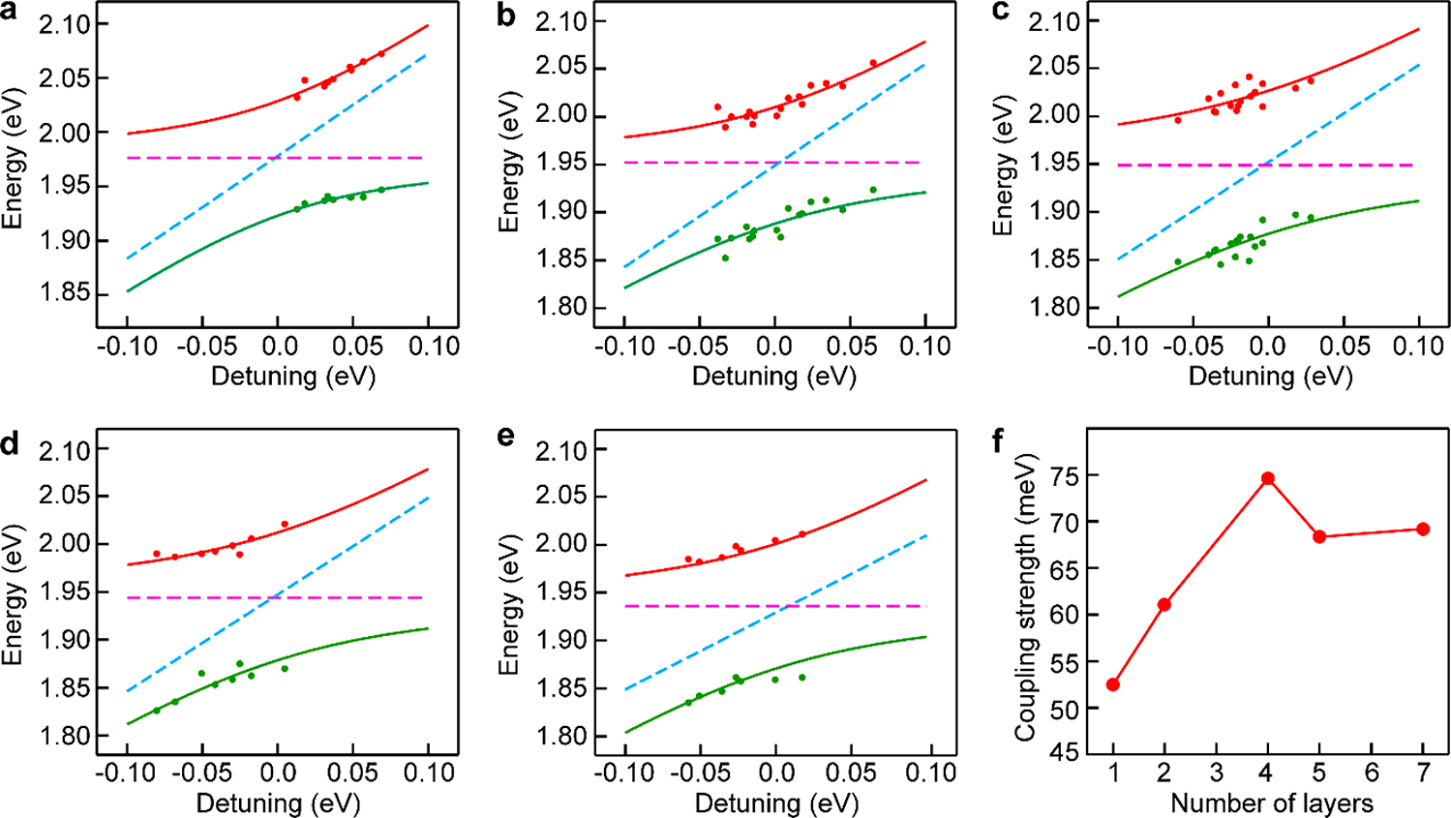
Plasmon–exciton coupling between the Au nanocups and WS_2_ monolayer/multilayers. (a–e) Resonance peak energies retrieved from the scattering measurements (red and green dots) as functions of the energy detuning between the plasmon resonance and the exciton transitions from 1-, 2-, 4-, 5-, and 7-layered WS_2_ nanosheets. The red and green solid lines were obtained from fitting based on the coupled oscillator model. The two dashed lines in magenta and blue denote the uncoupled exciton and plasmon energies, respectively. (f) Variation of the coupling strength with the WS_2_ layer number. The coupling strength *g* values were estimated from the plots in (a)–(e) using the coupled oscillator model.

We finally examined the interaction of the magnetic plasmon resonance of the Au nanocups with the excitons in WS_2_ multilayers. The Au nanocup sample 1 with the magnetic plasmon peak located at ∼606 nm was deposited on WS_2_ of different layer numbers (Figure S13). The numbers of the layers in the used WS_2_ nanosheets were determined with AFM (Figure S14). The absorption spectra of the downward-orientated Au nanocup on WS_2_ monolayer and multilayer were simulated (Figure S15). The dielectric functions of WS_2_ monolayer and multilayer were taken from previous works.^61,62^ The simulated absorption spectra of the hybrid system composed of WS_2_ multilayer show the same mode splitting behavior. As the layer number is increased, the exciton transitions shift to lower energies, leading to the redshifts of the dip in the scattering and absorption spectra (Figure S15).^37^ Implementing the same analysis as in the case for WS_2_ monolayer revealed the similar anticrossing behaviors in 2-, 4-, 5-, and 7-layered WS_2_ systems (Figure 5a–e). The coupling was found to strengthen with the layer number and become saturated at ∼4 layers (Figure 5f). The Rabi splitting energy in the 4-layered WS_2_ system exceeds both (γ_pl_ – γ_ex_)/2 and (γ_pl_ + γ_ex_)/2, implying that the system reaches the regime of strong coupling. When the number of WS_2_ layers is increased to much thicker flakes (5 and 7 layers), the coupling strength shows a slight decrease. To understand the experimental results, we calculated the electric field enhancement of the Au nanocup on WS_2_ monolayer (1 L), 4 layers (4 L), and 7 layers (7 L). Their thicknesses are 1.0, 3.0, and 5.0 nm, respectively. The anisotropic dielectric response of WS_2_ was not considered in the simulations due to the limited understanding of the out-of-plane dielectric function of WS_2_ monolayer and multilayer.^63^ Therefore, only in-plane electric enhancement (|*E*_*xy*_/*E*_0_|^2^) was considered in the simulations. The same aforementioned excitation configuration was also employed, where the incident light was perpendicular to the substrate and polarized in the *x* axis direction. Only the Au nanocup in the downward orientation was considered. For all electric field enhancement contours (Figure S16a), the *x*–*y* plane was taken from the top surface of WS_2_, with *z* = 0.0 nm. As the WS_2_ layer number is increased, the electric field enhancement in the top layer shows decreases (Figure S16a). The electric field enhancement profiles extracted on the *x*–*y* plane at *y* = 0.0 nm (Figure S16b) show the respective enhancement decays from *z* = 0.0 nm to *z* = 1.0 nm (1 L), 3.0 nm (4 L), and 5.0 nm (7 L). The step used in the simulations along the *z* direction was 0.5 nm. The maximal electric field enhancements in Figure S16b as a function of the distance in different WS_2_ layers (*z*, nm) are shown in Figure S16c. A slight decrease in the electric field enhancement is seen when the layer number of WS_2_ is increased to 4. When multilayered WS_2_ nanosheets are present, the coupling can involve a larger amount of excitonic material than that in the monolayer case. For the Au nanocup–monolayer system, the transition dipole of the exciton is oriented in the monolayer plane and can only interact with the *E*_*xy*_ field induced by the Au nanocup. The excitons in WS_2_ multilayer also have an out-of-plane component. The out-of-plane exciton component can also couple with the strong *E*_*z*_ field, resulting in an increase in the coupling strength.^34^ However, as the layer number is further increased, the overall electric field enhancement is reduced significantly (Figure S16c). The in-plane dipole moment of WS_2_ has been demonstrated to decrease with increasing WS_2_ layers.^64^ Although more excitons participate in the plasmon–exciton coupling, the reduced plasmonic electric field enhancement and the reduced exciton dipole moment are both responsible for the weakening and saturation of the observed coupling strengths at 5 layers and above. These results are in agreement with recent experimental works.^34,37,39^

## Conclusion

We have demonstrated that the magnetic plasmon resonance supported on Au nanocups can couple to the excitons in the WS_2_ monolayer, showing an orientation-dependent coupling behavior. Resonance coupling with the strength varying from the weak to strong regime is realized by adjusting the orientation and size of the individual Au nanocups on WS_2_ layers. Energy detuning between the magnetic plasmon resonance and the exciton transitions in WS_2_ monolayer is systematically varied, which gives rise to a mode splitting energy of 106 meV at zero detuning. The occurrence of mode splitting in the measured scattering and simulated absorption spectra suggests that the plasmon–exciton coupling in our hybrid system starts to enter the strong coupling regime. Moreover, when the Au nanocups are resonantly coupled with WS_2_ multilayers, the Rabi splitting energy increases with WS_2_ layers because of the involvement of more excitons in the coupling. The Rabi splitting energy reaches maximum at ∼4 WS_2_ layers and then becomes saturated. Our results provide a facile and promising route toward the design of a simple hybrid system out of individual Au nanocups and two-dimensional TMDCs to realize the different coupling regimes. Moreover, considering that highly asymmetric plasmonic nanocups can bend light^65^ and largely enhance second-harmonic generation,^41,66^ we foresee that there exist many opportunities for the use of Au nanocups to manipulate the emissions of different excitons in diverse two-dimensional materials for applications in optics, optoelectronics, and quantum technologies.

## Methods

### Gold Nanocup Preparation

A sacrificial templating method was employed to prepare the Au nanocup samples, as reported previously.^40^ First, PbS nanooctahedra were grown by a wet-chemistry method.^67^ During growth, CTAB (0.1 M, 2.57 mL), lead acetate (0.5 M, 2.04 mL), thioacetamide (0.5 M, 2.04 mL), and acetic acid (1.0 M, 4.10 mL) were added sequentially into water (34.26 mL), followed by heating at 80–90 °C for 8 h. The grown PbS nanooctahedra were collected by centrifugation at 5000–6000 rpm for 10 min. Second, the preprepared PbS nanoocthedron solution was injected into the overgrowth solution of Au. Each overgrowth solution was made in advance by sequentially adding HAuCl_4_ (0.01 M, 2 mL) and ascobic acid (0.1 M, 2 mL) into a CTAB solution (0.075 M, 40 mL). After that, the PbS nanooctahedron solutions with different amounts (110, 90, 80, 70, 60, and 50 μL) were injected respectively into the Au overgrowth solution. To avoid aggregation, the obtained solution was mixed and subjected under sonication for 5 min. It was then kept undisturbed at 35 °C in an oven for 2–3 h. The hybrid nanoparticles obtained from Au overgrowth were precipitated by centrifugation at 4000–5000 rpm for 10 min. After the careful removal of the supernatant, the obtained nanoparticles were redispersed in an aqueous CTAB solution (0.1 M, 20 mL). The etching of the templating PbS nanooctahedra was carried out by adding HCl (5.0 M, 1 mL) into the Au/PbS hybrid nanoparticle solution and then keeping the mixture solution in an air-bath shaker at 60 °C overnight. The etching solution was thereafter subjected under centrifugation at 4000–5000 rpm for 10 min to precipitate the obtained Au nanocups, which were finally redispersed in water (10 mL) for further use.

### WS_2_ Nanosheet Preparation

The WS_2_ nanosheets were synthesized using a previously described physical vapor deposition method.^46^ This method was carried out inside a quartz tube in a tube furnace. Solid WS_2_ powder was used as the source material, with a 300 nm thick oxide-coated Si wafer (Si/SiO_2_) functioning as the substrate. There were gas inlet and outlet on both sides of the quartz tube. Nitrogen was used as the carrier gas. The valves equipped at the two ends of the reaction tube controlled the N_2_ gas flow direction. The preparation of WS_2_ nanosheets typically included two steps. In the first step, the carrying N_2_ gas at 60–120 sccm was controlled in backward flow at the temperature ramp stage to avoid undesired provision of the WS_2_ vapor and thus inhibit unwanted WS_2_ growth. In the second step, when the furnace reached the target temperature of 1170–1190 °C, a forward N_2_ gas flow was generated to transport the WS_2_ vapor from the solid WS_2_ powder to the substrate and thus grow WS_2_ monolayer and multilayers. After 2–5 min, the substrate with grown WS_2_ nanosheets was pulled out to stop the growth process.

### FDTD Simulations

FDTD Solution 8.7 developed by Lumerical was used for the FDTD simulations in this work. The Au nanocup was modeled using a partially cut Au ellipsoid and a water/air octahedron formed from the same two pyramids of square bases. The water/air octahedron was superimposed on the cut Au ellipsoid. The size parameters acquired from the SEM images (Table S1) were used to model the Au nanocup samples 1–6. The dielectric function of Au was based on Jonson and Christy’s data. The surrounding medium was either water or air, with their refractive indexes set as 1.33 and 1.0, respectively. To model the individual hybrid structure, the Au nanocup was placed on WS_2_ nanosheets with thicknesses varied among 1.0 nm (1 L), 2.0 nm (2 L), 3.0 nm (4 L), 3.5 nm (5 L), and 5.0 nm (7 L). The WS_2_ nanosheets were further supported on a 300 nm thick SiO_2_ layer. The refractive index of SiO_2_ was taken as 1.45, and the dielectric functions of WS_2_ monolayer and multilayers were obtained from previous works.^61,62^ The gap distance between the nanocup and the WS_2_ nanosheet was assumed to be 1.0 nm to take into account the CATB layer adsorbed on the Au nanocup. For all simulations, a total-field scattered-field source covering a spectral range of 400–1000 nm was introduced into a box housing the individual structure. The mesh size used for simulating the scattering/absorption spectra was 1.0 nm. The hybrid structure and its surrounding space were divided into meshes of 0.5 nm in size for simulating the contours of electromagnetic field enhancement and charge distribution.

### Characterization

SEM images were taken on a scanning electron microscope (JEOL JSM-7800F, Schottky field emission) operated at 10 kV. Extinction spectra were taken on an ultraviolet/visible/near-infrared spectrophotometer (PerkinElmer Lambda 950). The used plastic cuvettes had an optical path length of 1.0 cm. The thicknesses of the WS_2_ nanosheet samples were measured with an atomic force microscope (Veeco Metrology system, model no. 920-006-101), which was operated at the contact mode in air with sharp Si_3_N_4_ tips (Bruker). Dark-field scattering spectra were measured at the single-particle level on an upright optical microscope (Olympus, BX60) equipped with a monochromator (Acton, SpectraPro 2360i), a charge-coupled device camera (Princeton Instruments, Pixis 400, cooled to −70 °C), and a quartz–tungsten–halogen lamp (100 W). Both the excitation with the white light and the collection of the scattered light from the individual nanoparticles relied on a dark-field objective, which had a magnification of 100 and a numerical aperture of 0.9.

## Supporting Information Available

Additional SEM images and extinction spectra of the nanocups; schematic illustrations of the models for the simulations; photograph of the setup for the preparation of WS_2_ monolayer and multilayers; additional characterization results of the WS_2_ nanosheets; scattering spectra of the Au nanocup samples on SiO_2_ substrates; additional FDTD simulation results; additional scattering and absorption spectra of the Au nanocup samples coupled with WS_2_ monolayer and multilayer; AFM results of the WS_2_ nanosheets (PDF)
