# Supplementary material for: Orientation-Dependent Interaction between the Magnetic Plasmons in Gold Nanocups and the Excitons in WS2 Monolayer and Multilayer
Source: ACS Nano. 2023 Jan 20;17(3):2356–67. doi: 10.1021/acsnano.2c09099 (PMC9933610; doi:10.1021/acsnano.2c09099)
Supplement: Supplementary file 1 — nn2c09099_si_001.pdf [file nn2c09099_si_001.pdf]

Supporting Information

Orientation-Dependent Interaction between the Magnetic Plasmons in Gold Nanocups and the Excitons in WS_2 Monolayer and Multilayer

Ruoqi Ai,[†,§] Xinyue Xia,[†,§] Han Zhang,[‡] Ka Kit Chui,[†] and Jianfang Wang*[,†]

[†]Department of Physics, The Chinese University of Hong Kong, Shatin, Hong Kong SAR, China
[‡]School of Materials Science and Engineering, Zhejiang Sci-Tech University, Hangzhou 310018, China
[§]These authors contributed equally to this work.
*Corresponding author. Email: jfwang@phy.cuhk.edu.hk

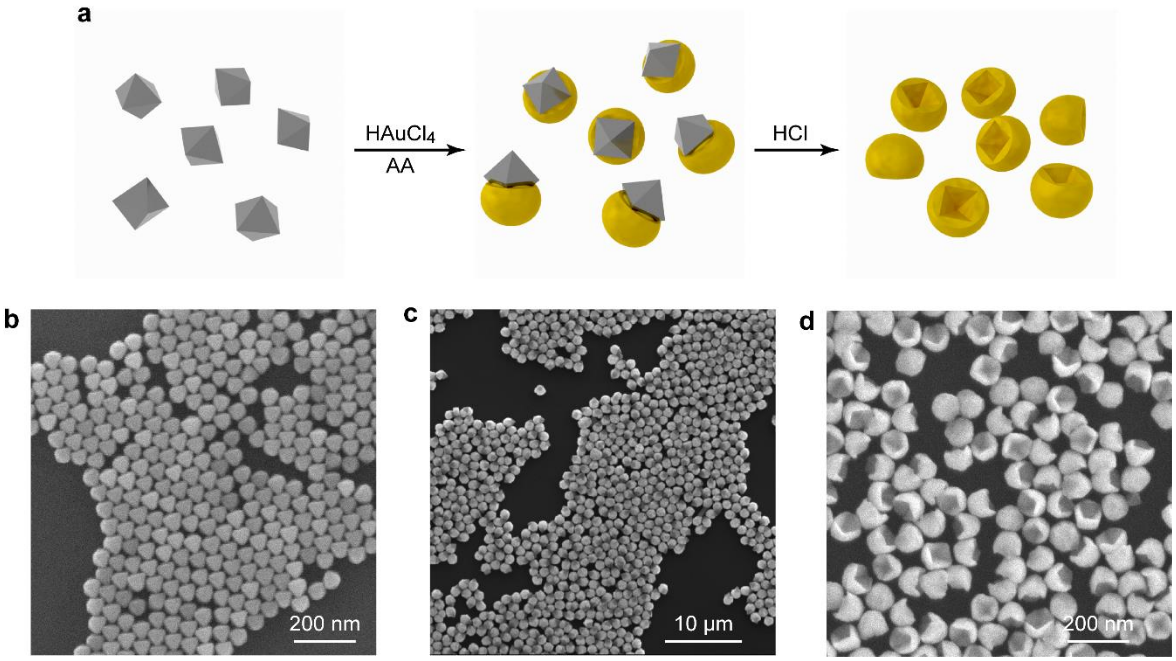

Figure S1. Synthesis of the Au nanocups. (a) Schematic illustrating the synthetic process. (b) SEM image of the PbS nanooctahedra. The edge length of the PbS nanooctahedra is 70 ± 2 nm. (c) SEM image of the Janus Au/PbS nanostructures. (d) SEM image of the representative Au nanocups.

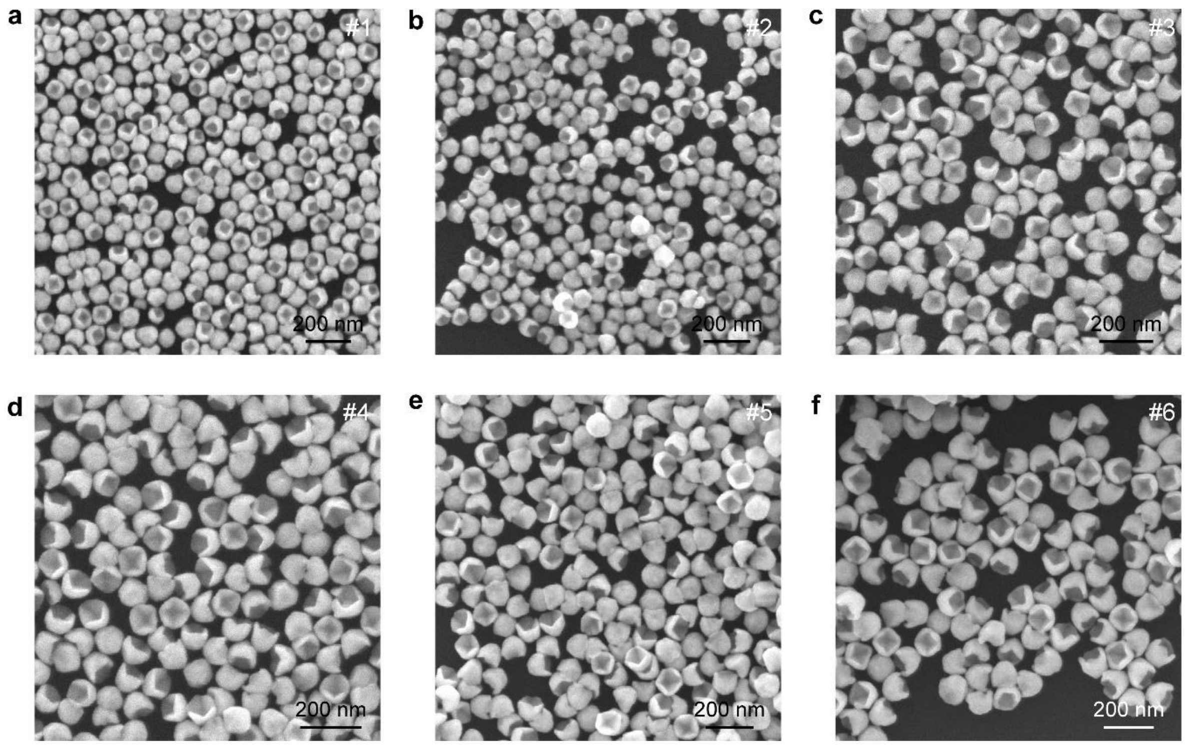

Figure S2. Representative SEM images of the six Au nanocup samples. The sizes of the Au nanocup samples increase from #1 to #6.

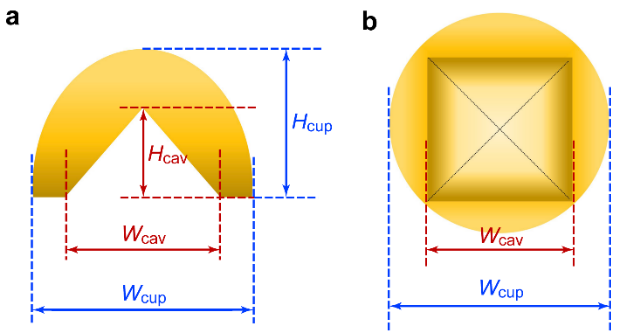

Figure S3. Model of the Au nanocup used in the simulations. (a) Viewed along the direction perpendicular to the symmetry axis of the Au nanocup. (b) Viewed toward the opening of the Au nanocup. The four size parameters used to describe the Au nanocup are indicated.

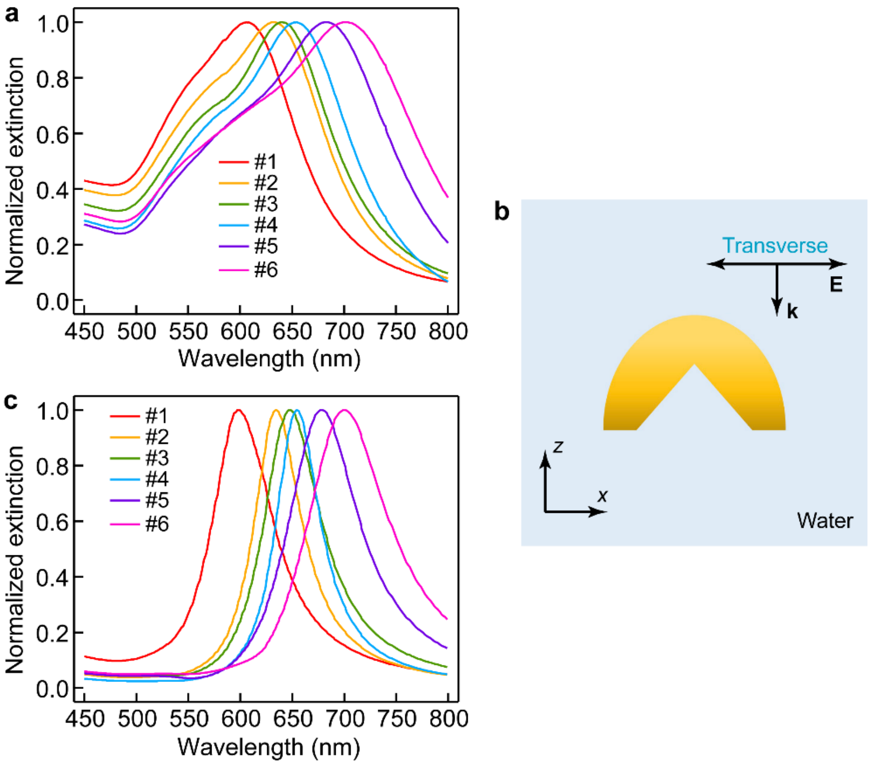

Figure S4. Plasmonic properties of the Au nanocup samples. (a) Measured extinction spectra of the six Au nanocup samples. The transverse resonance peak redshifts with increasing sizes. (b) Schematic of the excitation polarization configuration employed in the FDTD simulations. The excitation light is incident along the axial direction and polarized along the transverse direction. (c) Normalized simulated extinction spectra of the six averagely sized Au nanocups.

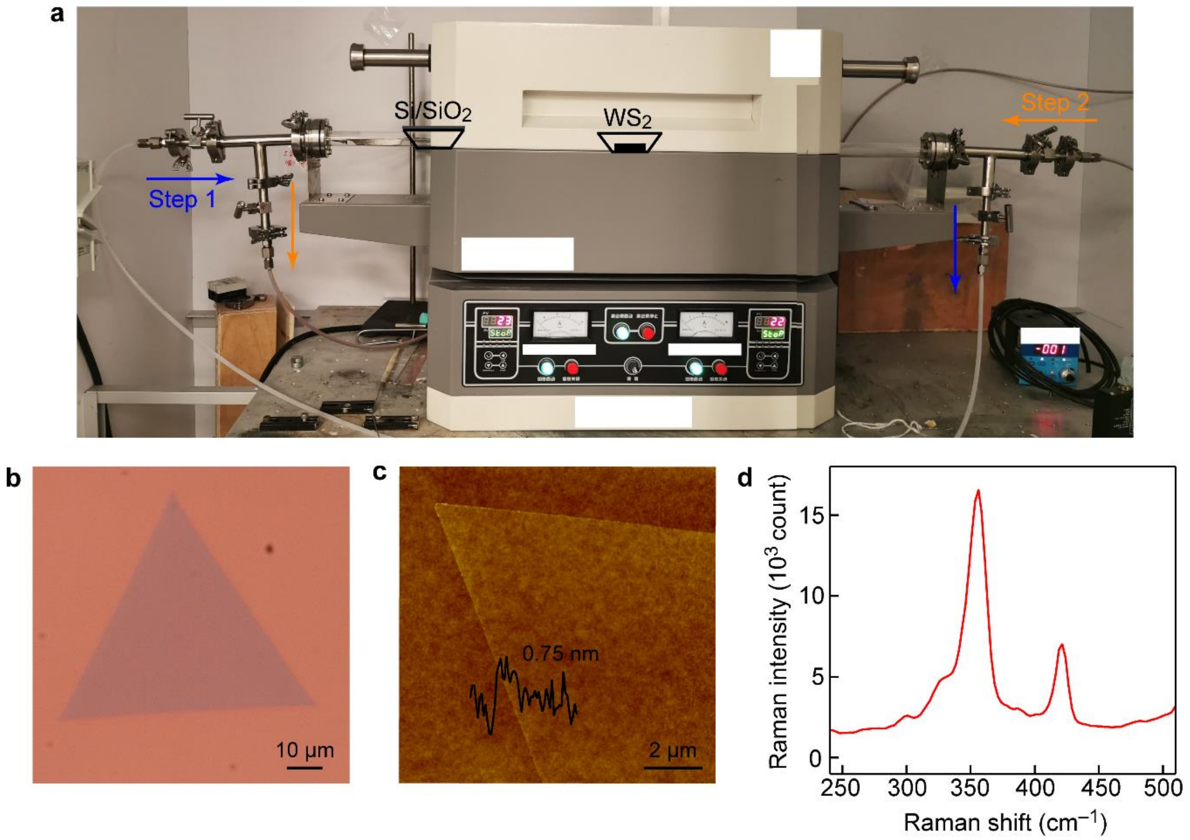

Figure S5. Preparation and characterization of the WS_2 nanosheets. (a) Photograph of the tube furnace used for the preparation of the WS_2 nanosheets. Step 1: the carrying N_2 gas was controlled in backward flow at the temperature ramp stage to prevent the unintended supply of the vapor source reactant or uncontrolled growth. Step 2: a forward flow from the source to the Si/SiO_2 substrate was applied to transport the vapor phase reactant onto the substrate for the growth of WS_2 monolayer and multilayer. (b) Bright-field optical microscopy image of a grown WS_2 nanosheet. (c) AFM image showing the thickness of the WS_2 monolayer. (d) Raman spectrum of the WS_2 monolayer. The optical power of the excitation laser at 488 nm was 0.225 mW, and the integration time was 1 s.

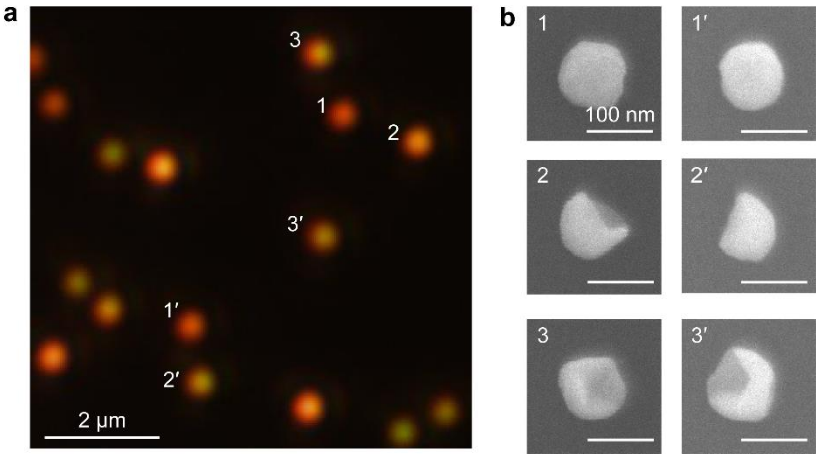

Figure S6. Au nanocups in different orientations on SiO_2 substrates. (a) Dark-field scattering image of the Au nanocups in different orientations on a SiO_2 substrate. (b) SEM images of the Au nanocups in the three typical orientations. 1 and 1′ are the Au nanocups with the downward orientation. 2 and 2′ are the Au nanocups with the sideward orientation. 3 and 3′ are the Au nanocups with the upward orientation.

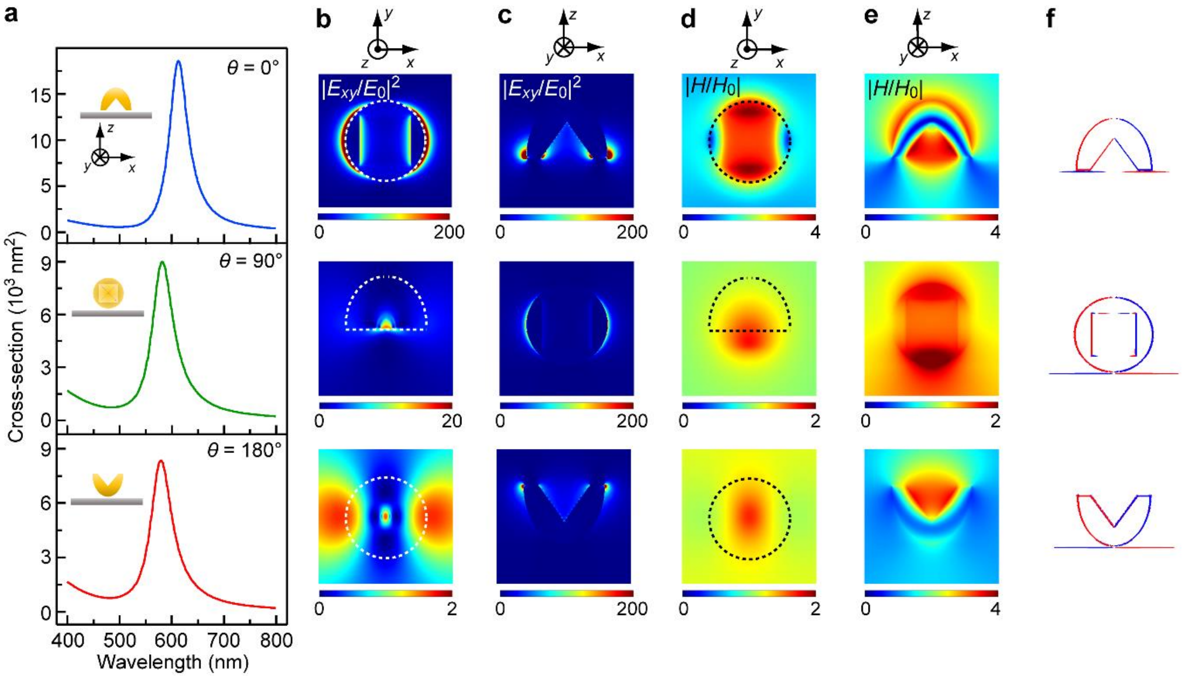

Figure S7. FDTD simulation of the Au nanocup in the different orientations on SiO_2 substrates. (a) Simulated scattering spectra of the Au nanocup in the three orientations on the SiO_2 substrate. The insets are the schematics of the Au nanocup in the three orientations. (b,c) Simulated electric field intensity enhancement contours at the peak wavelengths of 612 nm, 581 nm, and 578 nm from top to bottom in the *x*-*y* and *x*-*z* planes, respectively. The dashed lines indicate the Au nanocup. (d,e) Magnetic field enhancement contours at the peak wavelengths of 612 nm, 581 nm, and 578 nm from top to bottom in the *x*-*y* and *x*-*z* planes, respectively. (f) Charge distribution contours at the peak wavelengths of 612 nm, 581 nm, and 578 nm from top to bottom, respectively.

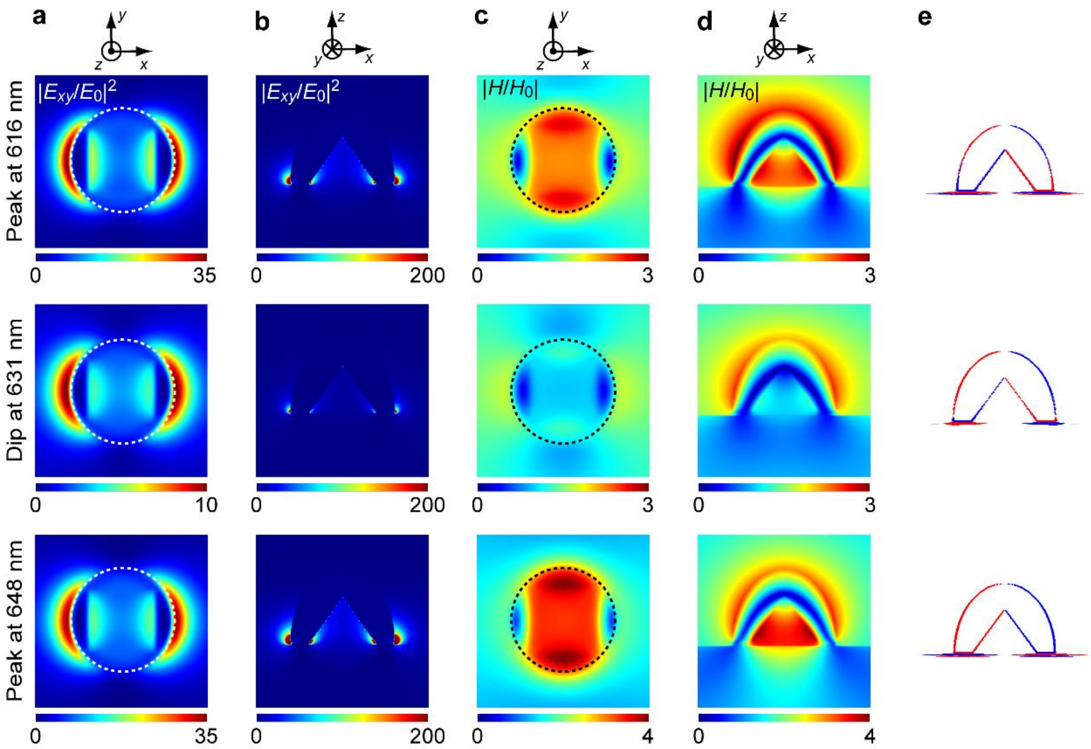

Figure S8. Simulation of the Au nanocup in the downward orientation on WS_2 monolayer. (a,b) Simulated electric field intensity enhancement contours at the wavelengths of 616 nm, 631 nm, and 648 nm from top to bottom in the *x-y* and *x-z* planes, respectively. The dashed lines indicate the surface of the Au nanocup. (c,d) Simulated magnetic field enhancement contours at the wavelengths of 616 nm, 631 nm, and 648 nm from top to bottom in the *x-y* and *x-z* planes, respectively. The dashed lines indicate the surface of the Au nanocup. (e) Charge distribution profiles at the wavelengths of 616 nm, 631 nm, and 648 nm from top to bottom.

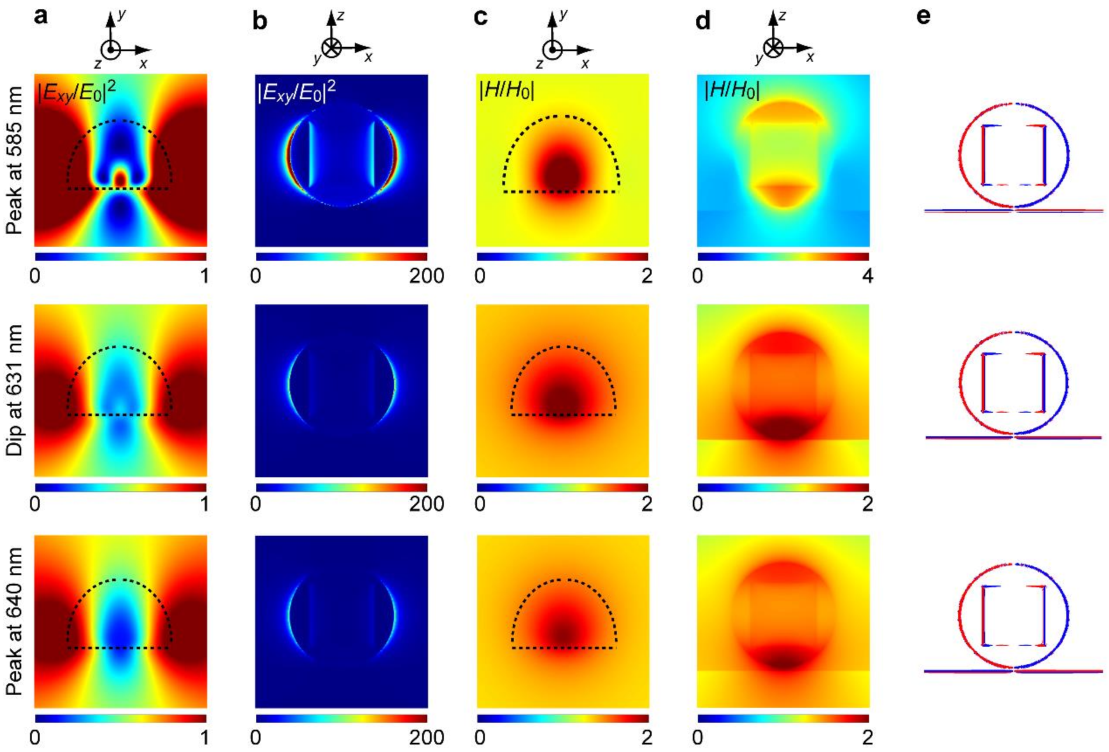

Figure S9. Simulation of the Au nanocup in the sideward orientation on WS_2 monolayer. (a,b) Simulated electric field intensity enhancement contours at the wavelengths of 585 nm, 631 nm, and 640 nm from top to bottom in the *x*-*y* and *x*-*z* planes, respectively. The dashed lines indicate the surface of the Au nanocup. (c,d) Simulated magnetic field enhancement contours at the wavelengths of 585 nm, 631 nm, and 640 nm from top to bottom in the *x*-*y* and *x*-*z* planes, respectively. The dashed lines indicate the surface of the Au nanocup. (e) Charge distribution profiles at the wavelengths of 585 nm, 631 nm, and 640 nm from top to bottom.

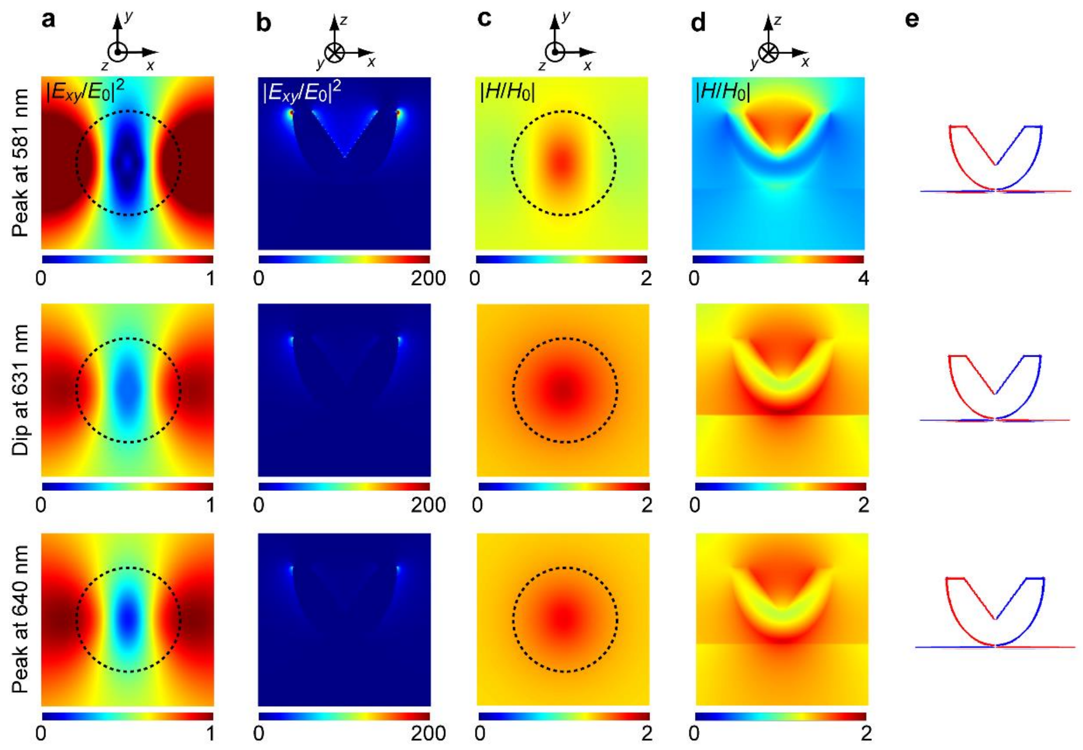

Figure S10. Simulation of the Au nanocup in the upward orientation on WS_2 monolayer. (a,b) Simulated electric field intensity enhancement contours at the wavelengths of 581 nm, 631 nm, and 640 nm from top to bottom in the *x*-*y* and *x*-*z* planes, respectively. The dashed lines indicate the surface of the Au nanocup. (c,d) Simulated magnetic field enhancement contours at the wavelength of 581 nm, 631 nm, and 640 nm from top to bottom in the *x*-*y* and *x*-*z* planes, respectively. The dashed lines indicate the surface of the Au nanocup. (e) Charge distribution profiles at the peak wavelength of 581 nm, 631 nm, and 640 nm from top to bottom.

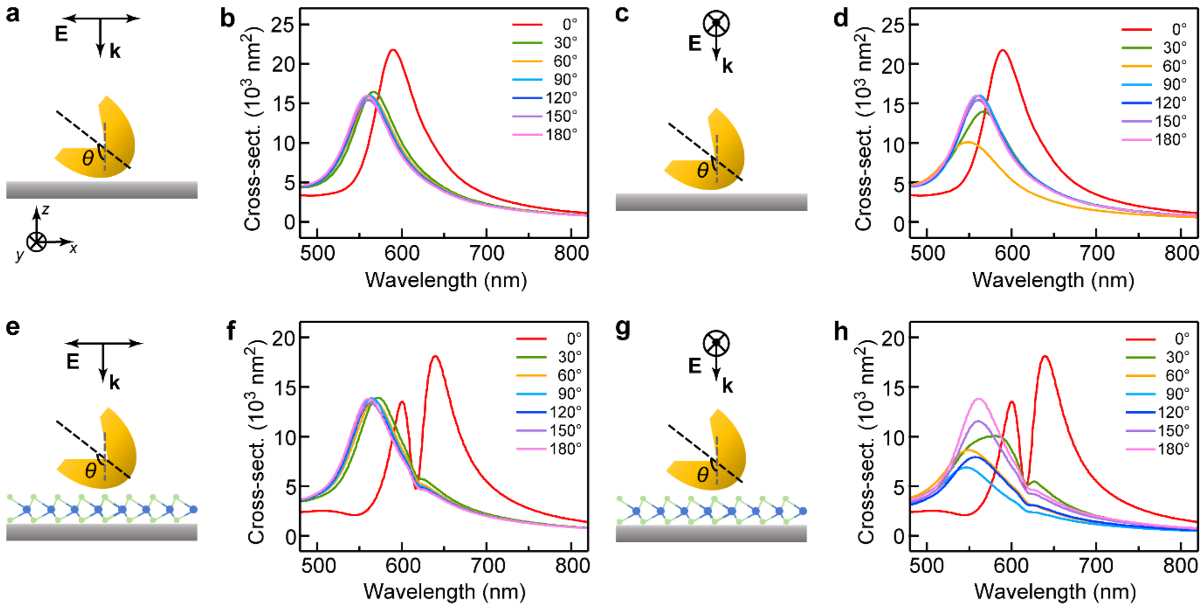

Figure S11. Effect of the nanocup orientation and the excitation polarization on the scattering spectra of the Au nanocup on the SiO_2 substrate and WS_2 monolayer. (a–d) Schematic and simulated scattering spectra of the Au nanocup on the SiO_2 substrate under *x*-polarized excitation (a,b) and *y*-polarized excitation (c,d). (e–h) Schematic and simulated scattering spectra of the Au nanocup coupled to WS_2 monolayer under *x*-polarized excitation (e,f) and *y*-polarized excitation (g,h).

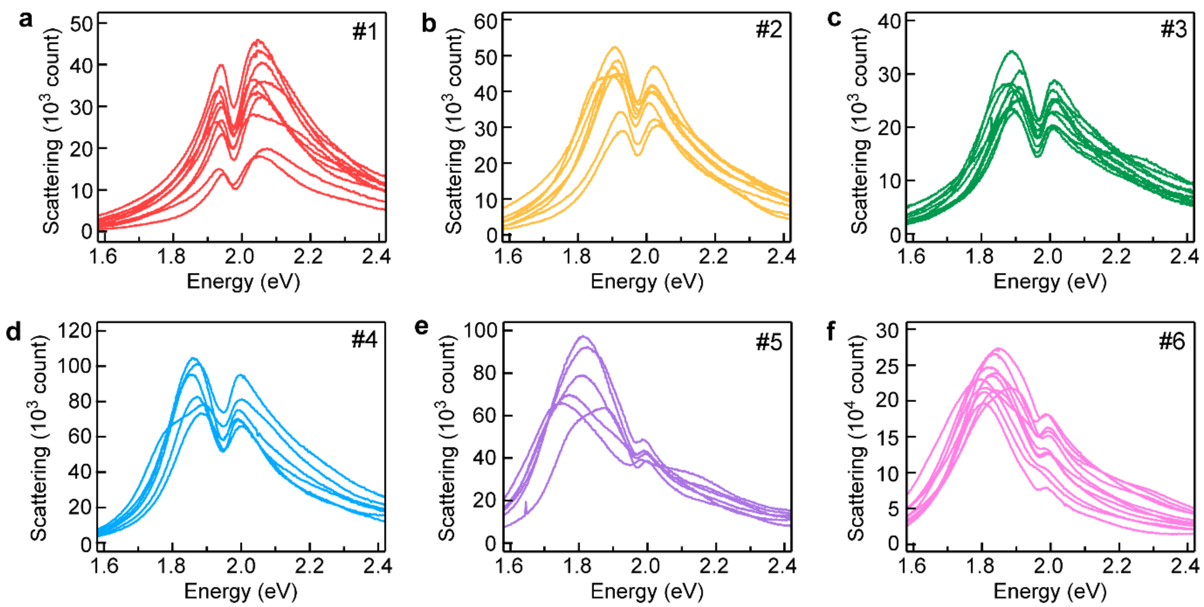

Figure S12. Differently sized Au nanocups coupled with WS_2 monolayer. (a–f) Scattering spectra of the representative Au nanocups from the samples #1–#6 coupled to WS_2 monolayer.

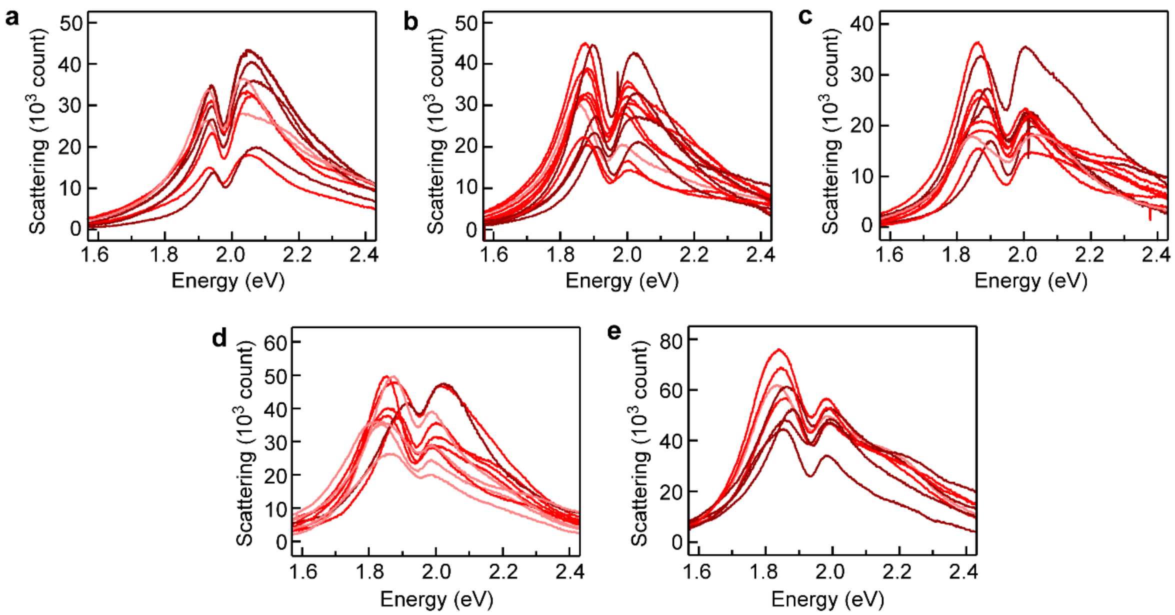

Figure S13. Au nanocups coupled with WS_2 nanosheets of different layer numbers. (a–e) Scattering spectra of the Au nanocups coupled with WS_2 nanosheets of different layer numbers. The Au nanocup sample #1 was used.

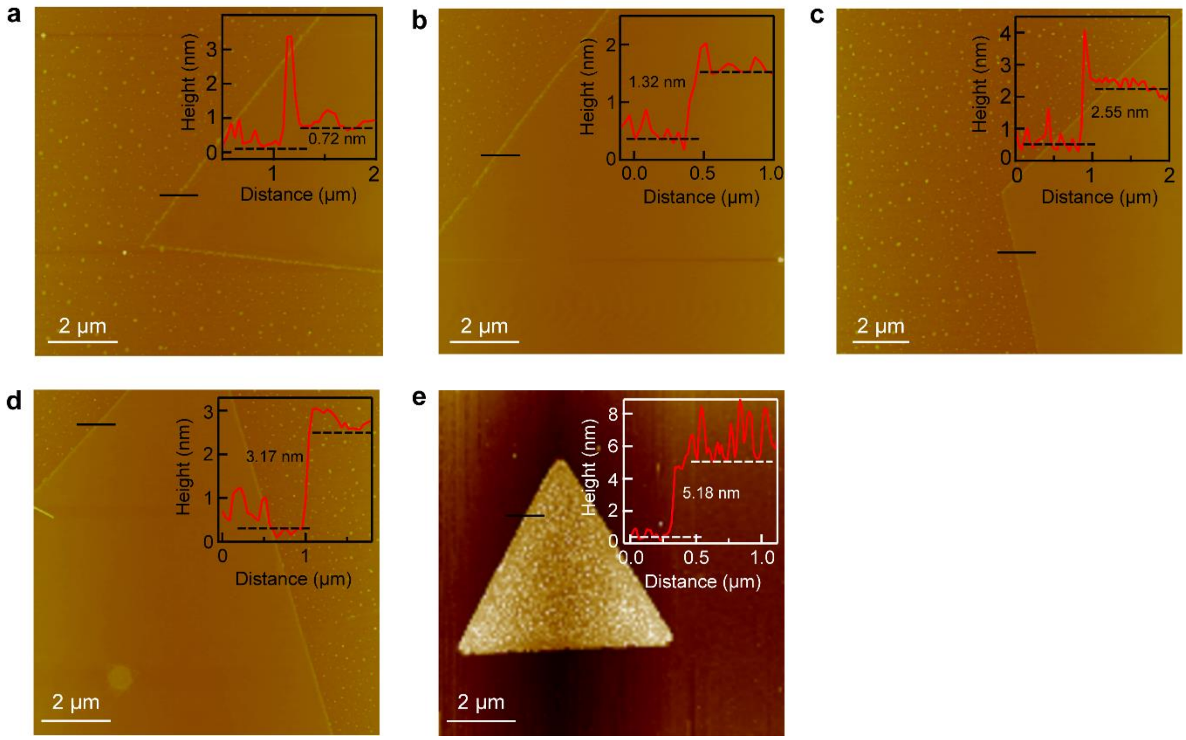

Figure S14. Characterization of the WS_2 nanosheets. (a–e) AFM images of the WS_2 nanosheets with 1, 2, 4, 5, and 7 layers, respectively. The insets are the height profiles exacted from the black line in each AFM image.

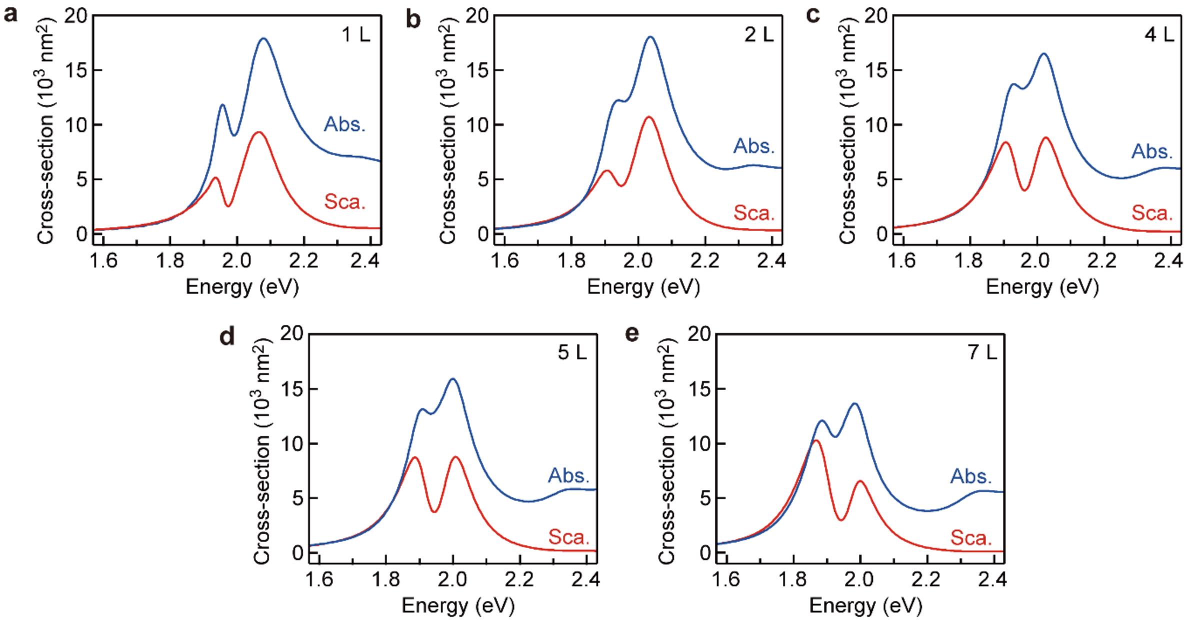

Figure S15. Simulated scattering and absorption spectra for the downward-oriented Au nanocup on WS_2 multilayer. (a–e) 1, 2, 4, 5, and 7 layers, respectively.

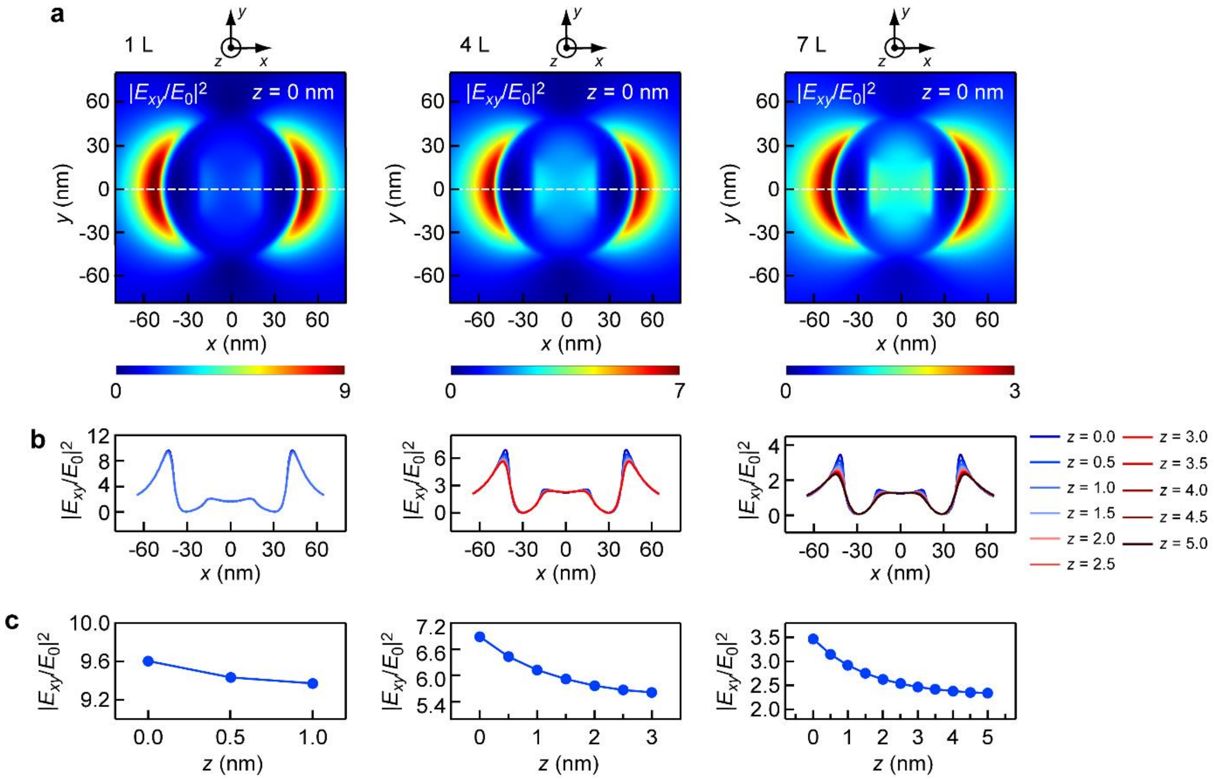

Figure S16. Electric field enhancement of the Au nanocup in downward orientation on WS_2 monolayer (1 L), 4 layers (4 L), and 7 layers (7 L). (a) Simulated electric field intensity enhancement contours of the Au nanocup coupled with WS_2 1 L, 4 L, and 7 L at the dip wavelengths in the scattering spectra. The dip wavelengths are 631 nm, 635 nm, and 643 nm from left to right, respectively. The white dashed lines indicate the positions where the electric field enhancement profiles were extracted. (b) Extracted electric field enhancement profiles in the *x*-*y* plane at *y* = 0.0 nm as a function of *z* increasing to 1.0 nm (1 L), 3.0 nm (4 L), and 5.0 nm (7 L) from left to right, respectively. The increasing step of 0.5 nm was used for simulating the electric field enhancement in the different WS_2 layers. (c) Maximal electric field enhancement decays as functions of the distance in the different WS_2 layers (*z*, nm). The layer number of WS_2 is 1, 4, and 7 from left to right, respectively.

Table S1. Measured Sizes of the Six Au Nanocup Samples and Corresponding Ones Used in the FDTD Simulations

measured (nm)	#1	#2	#3	#4	#5	#6
W_{cup}	85 ± 5	80 ± 5	84 ± 3	95 ± 5	105 ± 7	110 ± 5
H_{cup}	61 ± 7	60 ± 6	63 ± 4	77 ± 3	88 ± 5	95 ± 9
W_{cav}	39 ± 7	45 ± 3	58 ± 5	52 ± 4	68 ± 5	65 ± 5
used in the simulations (nm)	#1	#2	#3	#4	#5	#6
W_{cup}	85	85	85	95	105	115
H_{cup}	60	60	60	74	85	90
W_{cav}	35	45	55	55	70	61
H_{cav}	25	32	39	39	49	43
